# A paper-based, cell-free biosensor system for the detection of heavy metals and date rape drugs

**DOI:** 10.1371/journal.pone.0210940

**Published:** 2019-03-06

**Authors:** Alexander Gräwe, Anna Dreyer, Tobias Vornholt, Ursela Barteczko, Luzia Buchholz, Gila Drews, Uyen Linh Ho, Marta Eva Jackowski, Melissa Kracht, Janina Lüders, Tore Bleckwehl, Lukas Rositzka, Matthias Ruwe, Manuel Wittchen, Petra Lutter, Kristian Müller, Jörn Kalinowski

**Affiliations:** 1 Center for Biotechnology (CeBiTec), Microbial Genomics and Biotechnology, Bielefeld University, Bielefeld, Germany; 2 Cellular and Molecular Biotechnology, Faculty of Technology, Bielefeld University, Bielefeld, Germany; 3 Faculty of Biology, Mathematical Methods in Systems Biology, Proteome and Metabolome Research, Bielefeld University, Bielefeld, Germany; University of Lethbridge, CANADA

## Abstract

Biosensors have emerged as a valuable tool with high specificity and sensitivity for fast and reliable detection of hazardous substances in drinking water. Numerous substances have been addressed using synthetic biology approaches. However, many proposed biosensors are based on living, genetically modified organisms and are therefore limited in shelf life, usability and biosafety. We addressed these issues by the construction of an extensible, cell-free biosensor. Storage is possible through freeze drying on paper. Following the addition of an aqueous sample, a highly efficient cell-free protein synthesis (CFPS) reaction is initiated. Specific allosteric transcription factors modulate the expression of ‘superfolder’ green fluorescent protein (sfGFP) depending on the presence of the substance of interest. The resulting fluorescence intensities are analyzed with a conventional smartphone accompanied by simple and cheap light filters. An ordinary differential equitation (ODE) model of the biosensors was developed, which enabled prediction and optimization of performance. With an optimized cell-free biosensor based on the *Shigella flexneri* MerR transcriptional activator, detection of 6 μg/L Hg(II) ions in water was achieved. Furthermore, a completely new biosensor for the detection of gamma-hydroxybutyrate (GHB), a substance used as date-rape drug, was established by employing the naturally occurring transcriptional repressor BlcR from *Agrobacterium tumefaciens*.

## Introduction

Water quality assessment is an issue of global relevance. In regions that suffer from natural disasters or industrial accidents, for example, a quick and reliable test of water quality is indispensable to inhibit the spreading of diseases and to prevent human intoxications. Among the most widespread detrimental substances in water are heavy metals, in particular arsenic and mercury [1]. Mercury affects liver, kidney, and the central nervous system and may lead to severe diseases of these organs [2]. Mercury contamination of water causes steep health and human costs, as illustrated by the Minamata incidence, which lead to thousands of cases of methylmercury poisoning [3]. Hence, the detection of detrimental substances is a serious challenge of public health and environmental protection.

Specificity, sensitivity and speed are key features of suitable sensors. Biological sensing elements have the potential to meet these criteria and can even be superior to conventional chemical sensors. In addition, biosensors are applicable for a wide range of analytical purposes [4] and can potentially be produced at low costs [5]. Thus, biosensors are emerging as promising tools for water quality assessment.

Among others, a biosensor for mercury detection has been developed [6]. It is based on the MerR transcriptional activator protein, which is a member of the MerR family found in gram-negative bacteria [7]. In the presence of Hg(II) salts, MerR activates protein expression *via* the *mer* operator [8].

Besides natural and accidental water contamination, criminals deliberately add noxious substances to beverages to incapacitate their victims. Such date rape drugs cause symptoms such as a loss of consciousness and will [9]. Hereof, γ-hydroxybutyrate (GHB) and its precursor γ-butyrolactone (GBL) have been reported to be used in the majority of cases [10]. Trace amounts of GHB occur in the human body at concentrations of about 1 μg/mL as a component of the gamma-aminobutyric acid (GABA) pathway [10]. These amounts are negligible compared to the dose of GHB leading to incapacitation, which is approximately 2 g for an average adult [11]. The main challenge in uncovering GHB intoxication in cases of assault is the fast metabolic degradation of GHB, which occurs in the range of several hours [10]. Testing for GHB intoxication with high sensitivity (10 μg/mL in blood, 15 μg/mL in urine) is restricted to clinics that possess specialized equipment, for example HPLC or chemical analyzers [12]. A sensor that enables fast, reliable and robust point-of-care diagnosis with minimum amount of equipment and sample preparation remains to be developed.

**Fig 1:**
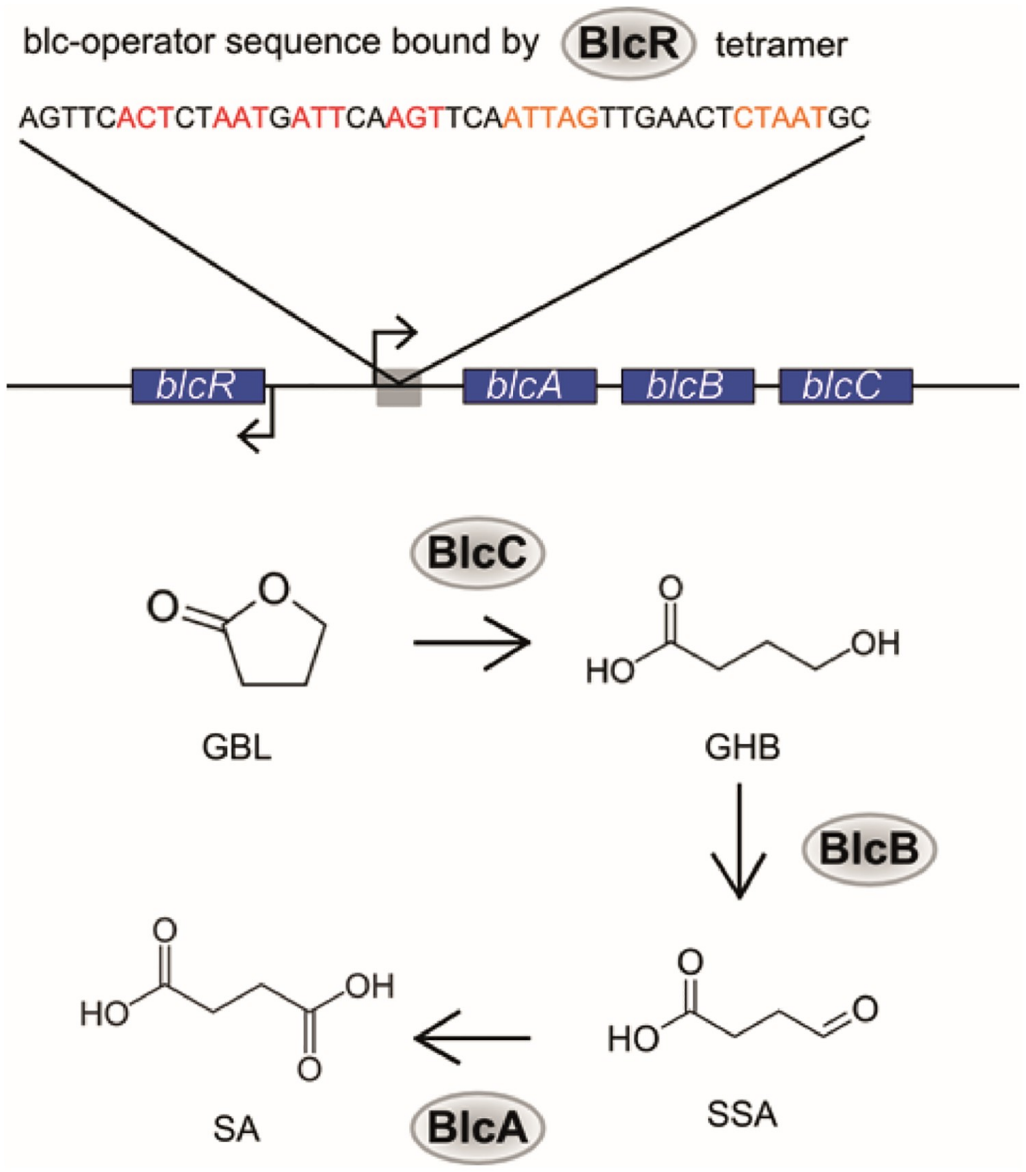
Schematic drawing of the *blc* operon and its corresponding pathway. The transcription of the GBL degrading *blc* operon in *A*. *tumefaciens* is controlled by the action of tetrameric BlcR [14]. The three proteins BlcC, BlcB and BlcA encoded in the operon enable *A*. *tumefaciens* to convert GBL into succinate [13]. GBL: γ-butyrolactone. GHB: γ-hydroxybutyrate. SSA: succinate semialdehyde; SA: succinate.

We identified a transcriptional repressor from *Agrobacterium tumefaciens*, BlcR, as a potential sensing element for GHB (Fig 1). BlcR naturally acts as a repressor of transcription by binding in tetrameric form to a DNA region upstream of the *blcABC* operon [13,14]. Upon binding of GBL or GHB, the BlcR tetramer releases its DNA recognition sequence and allows expression of the corresponding gene cluster [13,14]. The synthesized proteins BlcA, BlcB and BlcC enable *A*. *tumefaciens* to convert GBL into GHB and eventually into succinate, which enters the tricarboxylic acid cycle.

Ambitious efforts have been made towards the development of whole-cell biosensors. These use living cells for detection and have shown great potential, for example in heavy metal and DNA damage detection [15,16]. However, the application of living, genetically modified organisms outside the laboratory can be complicated by legal regulations and safety concerns. In addition, such whole-cell biosensors suffer from relatively short shelf lifes and long response times [5].

Synthetic *in vitro* biosensors overcome these problems [6,17]. In terms of biosafety, they are more appropriate for the open field, and can be optimized with regard to speed and specificity. Progress has been made towards easy-to-handle and cheap matrices and equipment [18,19], which brings biosensors closer to real-life application.

Using cell-free protein synthesis (CFPS), proteins are produced from nucleic acid templates without living cells. CFPS emerged as a valuable tool for various applications. With CFPS, the biological synthesis of toxic proteins became possible [20]. Other applications include the preparation of complex, native membrane proteins [21] and the incorporation of non-natural amino acids into proteins via *in vitro* translation with appropriate aminoacyl tRNA synthetases [22]. Furthermore, the use of *Escherichia coli* CFPS for diagnostic purposes has recently been described and simple paper as a cheap matrix was introduced [17,18].

CFPS systems can be adjusted by a change in component concentrations or the use of additional supplements while the reaction environment stays defined. The testing of various reaction conditions can be facilitated by mathematical modeling. Progress has been made towards full comprehension of CFPS. Especially the PURE system, a cell-free reaction environment completely made of purified components [23], has been extensively studied [24,25].

Here, we aimed to develop an easy-to-use biosensor system with high application potential regarding open-field usage. This was achieved by using naturally occurring allosteric transcription factors paired with CFPS. The biosensor output is based on the expression of sfGFP [26]. As sfGFP folds rapidly and remains stable under harsh conditions, it is an ideal reporter protein for *in vitro* protein synthesis [27,28]. To address the problem of fluorescence detection in the open field, the use of simple light filters and smartphones would be a user-friendly solution [29,30]. Consequently, a two-filter system in combination with a conventional smartphone was developed in this study. Specific excitation and emission wavelengths of the fluorophore were obtained via suitable light filters in front of the flash and the camera. Thus, the detection of sfGFP fluorescence without the need for expensive hardware was demonstrated. To minimize the background signal, the detection should be performed in a dark environment, provided by a black case. A 3D printer template of this case as well as a mobile application for the smartphone that simplifies fluorescence signal evaluation are presented (S1 and S2 Files).

Based on the model of the PURE system [24,25] and the data produced in this study, several CFPS conditions were simulated *in silico*, aiming to investigate the requirements for an optimal performance of cell-free biosensors. In particular, the influence of the allosteric transcription factor during the reaction was investigated, as this protein determines the detection limit of the biosensor. The extensibility of the biosensor was demonstrated by using two different transcription factors, the MerR protein and its *mer* operator to detect mercury and the BlcR protein and its operator from *A*. *tumefaciens* to detect GHB/GBL.

## Materials and methods

### General methods

Unless otherwise noted, *E*. *coli* was routinely grown at 37 °C and 200 rpm in LB medium with selective antibiotics. Plasmids were amplified in *E*. *coli* KRX competent cells (Promega). Detailed information about the strains and the primers used for cloning can be found at http://2015.igem.org/Team:Bielefeld-CeBiTec (hereafter referenced as wiki). Significance levels were calculated using Student’s independent two-sample *t*-test assuming equal variance.

### Construct assembly

Genetic constructs were assembled via restriction and ligation or Gibson assembly, as previously described by Gibson et. al. 2009 [31]. Successful assembly was verified via PCR and sequencing. Numbers in the format BBa_XXXXXXXX. correspond to BioBricks. Already existing BioBricks were provided by the iGEM headquarters (Boston). Sequences are available in the iGEM parts registry (http://parts.igem.org).

For optimized sfGFP (BBa_I746916) expression, a translation enhancing 5’-untranslated region (5’-UTR) based on work from Olins, Takahashi, Karig, Lentini and colleagues [32–35] was designed (BBa_K1758100, S1 Text). This 5’-UTR was cloned between P_T7_ and the sfGFP coding sequence via Gibson assembly, thereby generating BBa_K1758102.

Among other additives, an endogenous *E*. *coli* RNase E inhibitor protein, RraA, has been described to improve yields of CFPS reactions [36]. The *rraA* gene was amplified via PCR from *E*. *coli* K12 genomic DNA. *rraA* with N-terminal 6x-His-tag and TEV-protease cleavage site was cloned into pSB1C3 via Gibson Assembly, resulting in BBa_K1758122.

For the mercury biosensor, an *E*. *coli* codon optimized version of the *merR* sequence from the *Shigella flexneri* R100 plasmid Tn21, part of a mercury dependent operon [37], and its corresponding operator *merT* were synthesized (Integrated DNA Technologies, IDT). The construct BBa_K1758340 contains *merR* under the control of the constitutive promoter BBa_K608002 and was generated by Gibson assembly [31]. *MerT* was cloned into pSB1C3 under control of P_T7_ and upstream of 5’-UTR-sfGFP (BBa_K1758101), whereby BBa_K1758344 was generated.

For the date rape drug sensor, *blcR* and the *blc*-operator sequence were amplified from *A*. *tumefaciens* C58.1 via PCR. *blcR* was cloned into pSB1C3 under control of the constitutive promoter BBa_K608002, generating BBA_K1758370. The *blc* operator was cloned into pSB1C3 under control of P_T7_ and upstream of 5’-UTR-sfGFP (BBa_K1758101), resulting in BBa_K1758376BBa_K1758376. For *in vivo* studies, BBa_K1758370 and BBa_K1758376 were combined to BBa_K1758377 via 3-A-assembly into pSB1T3.

All *Spe*1, *Xba*1, *Eco*R1 and *Pst*1 restriction sites except for the ones from prefix and suffix for BioBrick Standard Assembly [38] were removed via mutagenesis PCR and all constructs were transferred to the plasmid backbone pSB1C3 to meet the requirements of the parts registry.

### Expression and purification of RraA

4x 200 mL LB with 20 μg/mL chloramphenicol in 1 L shake flasks were inoculated with an overnight culture of *E*. *coli* ER2566 carrying BBa_K1758122. Protein production was induced after 2 h with 5.5 μM L-rhamnose (Roth). Cells were harvested by centrifugation (37,500 x g, 4 °C, 30 min) 3 h after induction according to Gorna *et al*. [39]. The protein was purified using Protino Ni-TED kit from Macherey-Nagel and concentrated using Vivaspin columns with 10 kDa cut-off (MerckMillipore). The effect of RraA protein on CFPS was investigated by supplementing it at 0.3 mg/mL final concentration to the cell-free reaction.

### *In vivo* studies

The inherent tolerance of *E*. *coli* KRX towards GHB and GBL (Roth) was tested and 1% (v/v) of GHB (approximately 108 mM) and 3% (v/v) of GBL (approximately 324 mM) supplied to LB medium were determined to be tolerable for bacterial growth. To observe the effect of BlcR *in vivo*, an *E*. *coli* KRX strain carrying the reporter construct BBa_K1758377 was used. Cultures were supplemented with 0.2% or 1% (v/v) of GBL and GHB, respectively, and protein expression via P_T7_ was induced by addition of 5.5 μM L-rhamnose at OD_600_ = 0.7–0.8. The fluorescence signal of sfGFP in 50 μL of each culture was monitored with a TECAN infinite M200 plate reader right before and 7 h after induction, at an excitation wavelength of 480 nm and an emission wavelength of 515 nm. Triplicates of each culture were measured and normalized to OD_600_.

### *In vitro* studies

For normalization, a cell lysate with sfGFP was produced. A 200 mL *E*. *coli* KRX culture harboring sfGFP under control of P_T7_ (BBa_I746916) was grown over night in the presence of 5.5 μM L-rhamnose. Cells were harvested, disrupted via sonication, and centrifuged for 30 min at 10,000 x g. The fluorescence signal of the supernatant was used for normalization of *in vitro* sfGFP production.

*E*. *coli* KRX strains harboring BBa_K1758340 for constitutive expression of MerR and BBa_K1758370 for constitutive expression of BlcR, respectively, were grown at 37 °C at 300 rpm in 2 L shake flasks containing 500 mL 2xYT+P medium (22 mM KH_2_PO_4_, 40 mM K_2_HPO_4_, 16 g/L tryptone, 10 g/L yeast extract, 5 g/L NaCl. All chemicals were from Roth). Cell extract for cell free protein synthesis was prepared as described below.

### Cell extract preparation

Cell extract was prepared according to published protocols [28] with minor modifications. For standard extract, *E*. *coli*ER2566 was plated on LB agar and cultivated in an 37 °C incubator for approximately 16 h. Subsequently, 4 mL 2xYT+P medium were inoculated with a single colony and incubated for 10 h, transferred to 100 mL 2xYT+P and cultivated for 10 h at 37 °C and 180 rpm. 50 mL of this preculture were used for inoculation of 5 L 2xYT+P in a 7 L stirred reactor (Biostat NLF) with an initial OD_600_ of approximately 0.15. Protein expression was induced with 1 mM IPTG at OD_600_ ≈ 0.9. Cells were harvested in mid-log phase to obtain highly active ribosomes, washed twice with 10 mL ice-cold S30A buffer (14 mM Mg-glutamate (Sigma), 60 mM K-glutamate (Sigma), 50 mM TRIS (Roth), 2 mM DTT (Roth), pH 7.7) per gram wet cells and once with S30 buffer (14 mM Mg-glutamate, 60 mM K-glutamate, 10 mM TRIS, 2 mM DTT, pH = 8.2). Pellets were flash frozen in liquid nitrogen and stored at -80 °C until further use. Cells were then thawed and resuspended in 1 mL S30 buffer per gram pellet, disrupted by sonication in 1.5 mL aliquots using a Bandelin HF-Generator GM 2070 in combination with an ultrasonic converter UW 2070, standard horn SH 70G, and microtip MS73. The lysate was centrifuged for 10 min at 12,000 x g and 4 °C. A run-off reaction for degradation of DNA was performed for 30 min at 300 rpm, 37°C in a thermomixer prior to a final centrifugation step for 10 min at 10,000 x g and 4 °C. Cell extract was flash frozen and stored at -80 °C.

### Conduction of a CFPS reaction in solution

For optimal planning of CFPS reactions, an excel spreadsheet similar to the one from Sun *et al*. [40] was set up. The composition of a standard CFPS reaction without DNA template is outlined in S1 Table. For reliable performance, premixes and mastermixes were prepared. Similar to Sun *et al*. [40], a loss of 0.75 μL by pipetting was assumed regarding one reaction with 15 μL. *E*. *coli* cell extract comprised 33% (v/v) of the final reaction volume. Unless otherwise noted, 10 nM of template DNA was used in each cell-free reaction.

After adding the mastermix to the DNA template, the sample was briefly mixed and centrifuged for 30 s at 10,000 x g at room temperature (RT). The reaction was conducted in black 384 flat bottom well plates (Corning). Fluorescence was monitored with a TECAN infinite M200 plate reader using Tecan i-control (1.10.4.0) in kinetic measurement mode at an excitation wavelength of 480 nm and an emission wavelength of 515 nm. One kinetic cycle consisted of linear shaking (amplitude 1 mm) for 3 s, waiting for 8 s, fluorescence detection (25 flashes per well), and 90 s waiting. Fluorescence of the mastermix without DNA was subtracted from the corresponding reactions, and subsequently values were normalized to cell lysate with sfGFP.

### Conduction of a CFPS reaction on paper

15 μL of final sample consisting of mastermix and DNA were applied onto autoclaved hole-punch paper discs with a diameter of 6 mm. For storage tests, the paper discs were put into reaction tubes, flash frozen in liquid nitrogen and directly lyophilized (Christ Alpha 1–4 LD plus). Lyophilization was conducted for 3 h. In preliminary work paper from different suppliers was tested, and C350L from Munktell proved to be the most suitable for the application. Paper discs were rehydrated immediately or after storage at 8 °C with 15 μL water to perform protein expression. The reactions were performed at 37 °C in black 96 flat bottom well plates (Corning). Fluorescence measurements and data processing were done as described before for CFPS reactions in solution.

### Modeling

The deterministic model of a CFPS-based biosensor was established with the software package SimBiology (Mathworks). The ordinary differential equations and parameters are available in the supplementary materials (S2 and S3 Tables, S1 Appendix). Most parameters were obtained from literature. To accurately describe the protein expression dynamics in our CPFS reactions, the Michealis-Menten constant of translation as well as the degradation parameters and the initial concentration of the TL resources, a species comprising the components required for translation, were determined by fitting to experimental data using the nlinfit (nonlinear least-squares problem) algorithm. The data set consisted of sfGFP expression profiles at various plasmid concentrations in a CFPS reaction (S3A Fig). Simulations were performed using the ode15s solver and varying the initial concentrations of crucial species (repressor gene, repressor dimer, reporter gene and analyte). The initial concentrations of all other species except for the TL resources were set to zero.

### Output signal processing

We designed a black case for fluorescence detection that was realized by 3D printing. Its exchangeable top is optimized for the Samsung Galaxy S5 mini smartphone (S1 File). It ensures reproducible conditions for imaging the paper-based biosensor in the lower drawer and hinders movement of the light filters. Various filters of a color filter catalog were tested for detection of sfGFP fluorescence (Lee Filters [41]). The filter Tokyo Blue (#071) in front of the flash combined with Twickenham Green (#736) in front of the camera generated the lowest background signal. A smartphone application was programmed using Android Studio 1.2.2 for Android 4.2 or higher (S2 File). It analyzes the green values of the spots with CFPS reactions and determines whether an analyte was present or not.

## Results and discussion

### Improvement of the cell-free reaction for biosensor purposes

A high signal-to-noise ratio is critical for biosensors. Hence, cell-free protein synthesis reactions were optimized to yield high amounts of sfGFP reporter protein.

Ribosomal translation is in general the major bottleneck of efficient protein expression in CFPS [25] and a variety of expression tags to improve protein yields have been developed [33,34,42,43]. A new translation enhancing sequence, 5’-UTR (S1 Text), was integrated into the P_T7_-sfGFP vector, downstream of the T7-promoter and upstream of *sfGFP*to improve overall sfGFP expression. The effect of this synthetic 5’-UTR on protein production was evaluated *in vivo* as well as *in vitro*.

**Fig 2:**
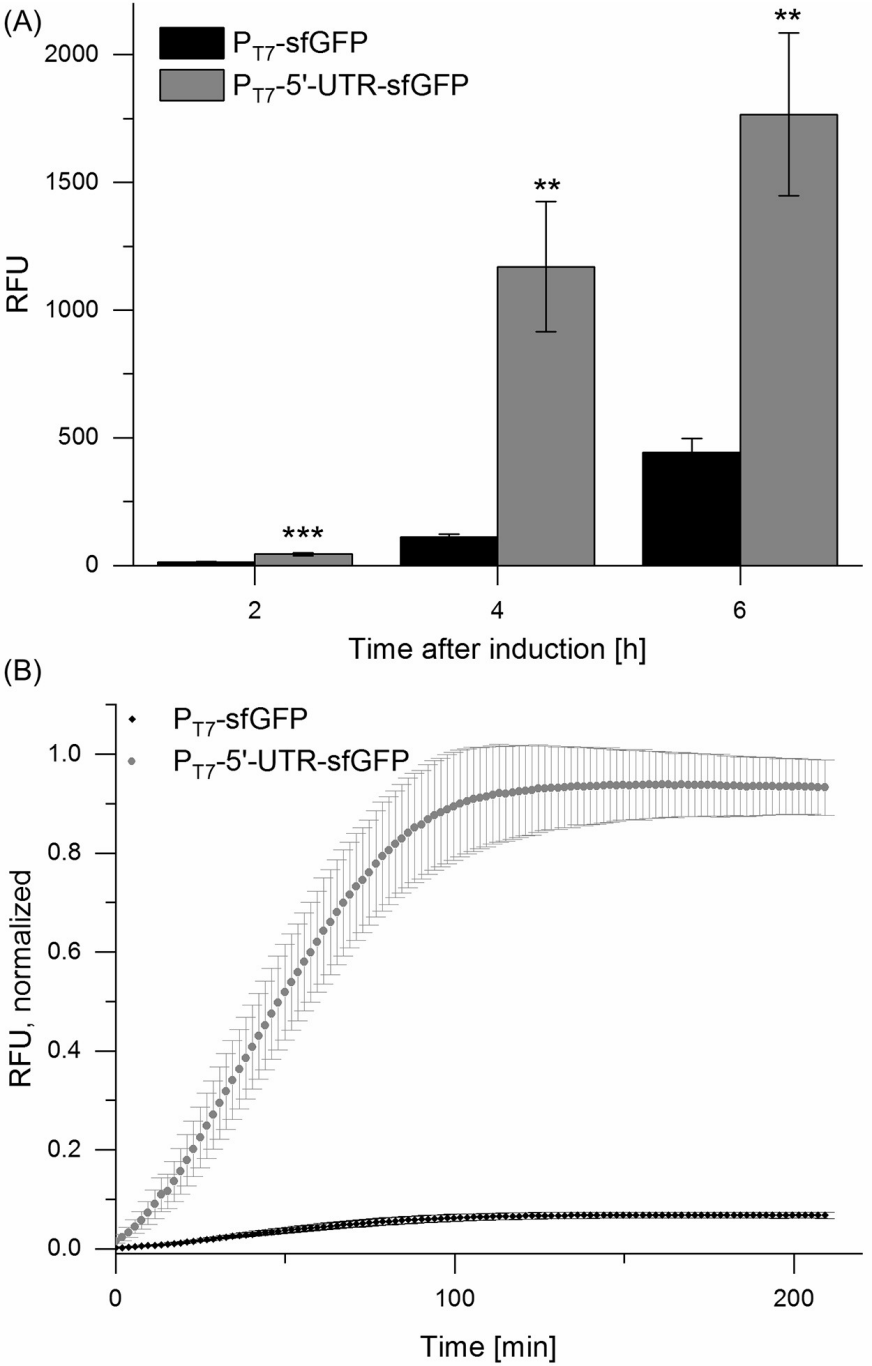
Enhancement of sfGFP expression by employing 5‘-UTR *in vivo* (A) and *in vitro* (B). (A) Fluorescence was normalized to OD_600_; (B) Fluorescence was normalized to a cell lysate with sfGFP. In both cases incorporation of the 5’-UTR into the P_T7_-sfGFP vector resulted in an increase of the fluorescence signal. Error bars represent the standard deviation of biological triplicates. **: p < 0.01; ***: p < 0.001.

The implementation of the synthetic 5’-UTR enabled improved expression of sfGFP compared to expression levels of sfGFP lacking the 5’-UTR (Fig 2). Especially for the *in vitro* expression of sfGFP the constructed 5’-UTR was crucial and protein yields were increased by a factor of 15, resulting in a final sfGFP concentration of approximately 230 μg/mL. This experimental setting for the expression of sfGFP, lacking any upstream repressor or activator site, was used as a positive control for subsequent *in vitro* experiments. Homemade CFPS worked well at 29 °C and 25 °C, although an incubation temperature of 37 °C yielded the highest fluorescence signal (S1 Fig). Applicability at ambient temperatures is an important step towards open-field usage.

Among other additives, an endogenous *E*. *coli* RNase E inhibitor protein, RraA, has been described to improve yields of CFPS reactions [36]. As RNase contamination is a well-known problem threatening CFPS reactions, the effect of RraA addition to our reaction was tested as an alternative to expensive RNase inhibitors. Purified RraA was added to the cell-free reaction at a final concentration of 0.3 mg/mL. The fluorescence signal after 2.5 h was enhanced by approximately 33% compared to reactions without RraA (S2 Fig), confirming the observations of Airen [36]. This highlights a major advantage of cell-free approaches over cell-based systems, namely the versatility and easy manipulation of the reaction composition.

### Modeling of CFPS-based biosensors

To develop a better understanding of the biological system and to investigate the influence of certain design aspects, we developed a mathematical model of CFPS-based biosensors.

#### CFPS model

Initially, a model was built to describe the cell-free expression of sfGFP. This model includes transcription and translation reactions, which were described using Hill kinetics. After its translation, sfGFP requires a maturation step in which the chromophore is converted into its active form [26,44]. This reaction was modeled using mass action kinetics. sfGFP degradation was neglected due to its high stability [26].

Data showed that the fluorescence signal reaches a plateau after one to three hours of a CFPS reaction. This cessation of protein synthesis in a batch-mode CFPS can be due to depletion of energy resources or amino acids [45,46], degradation of ribosomes [25], accumulation of inhibitory byproducts [47] or an unfavorable pH shift [48]. Although the reasons can be manifold, translation has unanimously been reported to be the limiting step, whereas transcription proceeds when protein synthesis has already stopped [25,49]. However, it is an important question whether translation stops because resources have been consumed by the translation reaction or due to a process that is not directly driven by translation, such as the degradation of ribosomes. Several experiments were performed to investigate this issue (similar to Stögbauer et. al. [25]). These indicated that the termination of protein synthesis is primarily a function of time and not dependent on the concentration of individual components. Consequently, a species named "TL resources" that catalyzes the translation reaction and degrades over time was incorporated into the model [25]. This species comprises all components that are necessary for translation. Its degradation, which was modeled using Michaelis-Menten kinetics, could be caused by several processes, such as enzymatic activity or a pH shift. The parameters were adopted from the literature or determined by fitting to experimental data. Comparing the model predictions to experimental data resulted in a good match (S2 and S3 Tables, S3 Fig).

#### Biosensor model

The CFPS model was subsequently expanded to describe a repressor-based biosensor. The dimerization, repression and derepression reactions and parameters were adopted from the *E*. *coli lac* operon model by Stamatakis and Mantzaris [50] and combined with the CFPS model described above. Transcription and translation of the repressor were described with the differential equations and parameters that had been determined for sfGFP. This approximation requires a similar plasmid design and comparable translation and folding efficiencies of the proteins.

**Fig 3:**
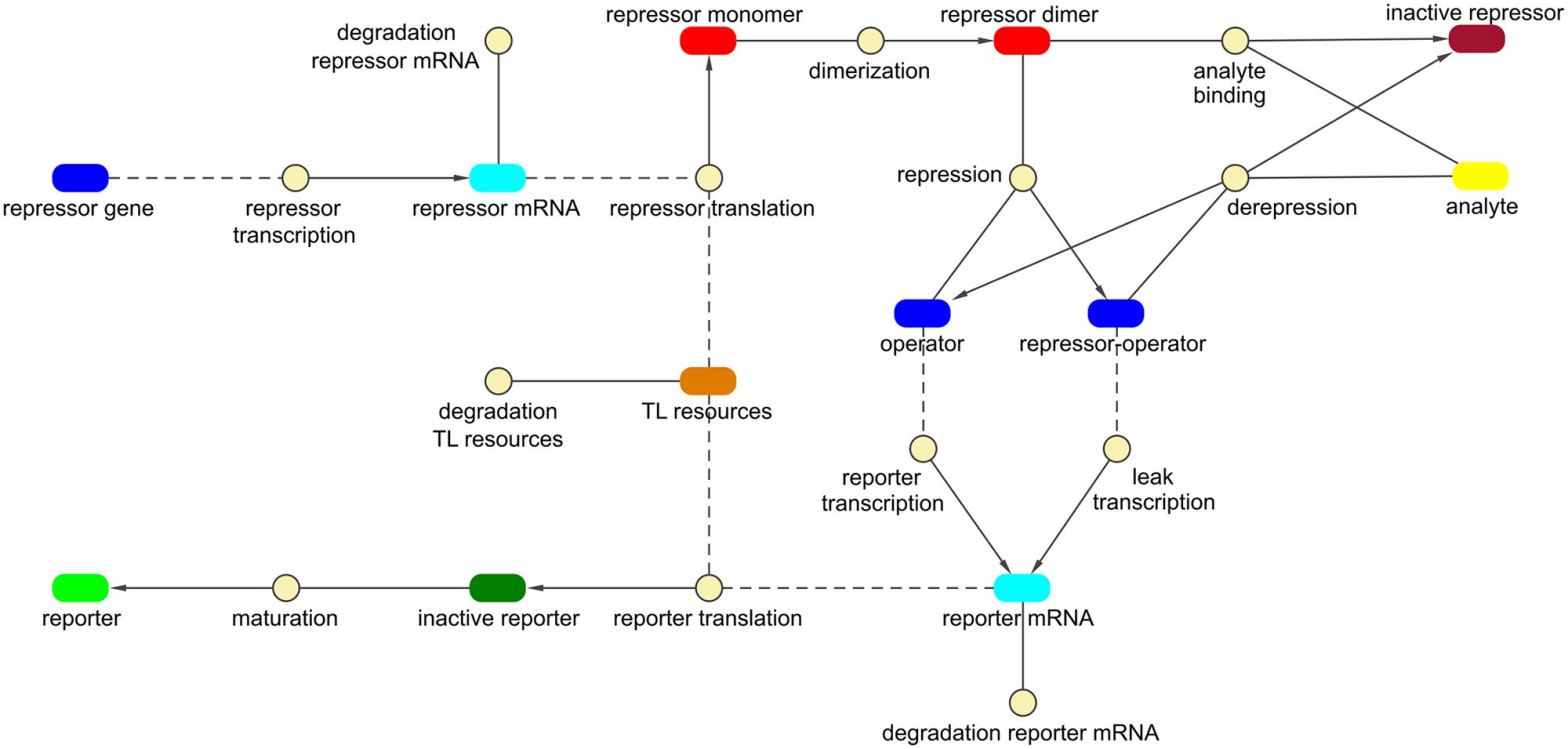
Illustration of the biosensor model. A model of the expression of a repressor-controlled reporter protein by CFPS was established. The rounded rectangles represent species such as DNA and proteins and the circles represent reactions. Lines indicate that a species is an educt in a reaction, arrows show that a species is the product of a reaction. Dashed lines mean that the species takes part in a reaction but is not consumed by it.

In experiments with various plasmid concentrations, it was observed that the protein synthesis capacity of a CFPS reaction is limited, resulting in a non-linear relationship between gene dosage and protein concentration. The extended model describes the expression of two genes, which means that their competition for the limited resources (such as ribosomes) had to be considered. This competition of reporter and repressor mRNA for translation resources was modeled as a competitive inhibition [51]. Fig 3 gives an overview of all species and reactions that were incorporated into the model. The differential equations and parameters are available in the supplementary materials (S2 and S3 Tables, S1 Appendix).

The completed model was used to simulate various scenarios. In particular, it can be used to illustrate the influence of the repressor protein, which is a crucial component of a CFPS biosensor as its concentration is decisive for the sensitivity and signal-to-noise ratio of the biosensor. There are two fundamentally different scenarios with regard to the repressor: It can be either encoded on a plasmid and be co-expressed with the reporter or it can already be present in the reaction mixture as a pre-expressed protein (S4B Fig). Co-expressing reporter and repressor is a convenient method for producing a biosensor. The model shows that the ratio of the plasmid concentrations is very important in this case (S4A Fig). If the reporter-to-repressor ratio is too high, a strong background signal occurs and the relative increase in the presence of an analyte is small and would be difficult to discern. In contrast, a low ratio would inhibit an induction due to strong repression and a scarcity of resources, which are consumed by the expression of the repressor. Given the parameters of this model a plasmid ratio of 1:1 yields a good compromise between low background and strong signal upon induction (S4A Fig).

However, the model demonstrates that pre-expression of the repressor in cells yields better results in terms of signal intensity and background noise, as the competition for resources does not limit the strength of the output signal and a repression may occur right from the beginning when no analyte is present. Consequently, cell extracts that already contained the necessary repressor or activator protein were used for all biosensor experiments (S4B Fig).

### Paper-based CFPS with homemade extract

It has been shown that CFPS reactions work under a wide range of conditions. Importantly, the use of paper as matrix for cell-free reactions is possible and holds huge potential for diagnostic purposes [18,52]. CFPS reactions with the optimized sfGFP template under the control of the T7 promoter (BBa_K1758101) were transferred onto paper discs and incubated at 37 °C to verify the feasibility of on-paper protein synthesis with self-made extracts. Various paper types were screened and in all cases it was crucial to autoclave the paper to obtain fluorescence signals comparable to CFPS reactions in solution. Blotting Paper C350L (Munktell) was chosen because high fluorescence levels with little variation were observed in these preliminary experiments.

**Fig 4:**
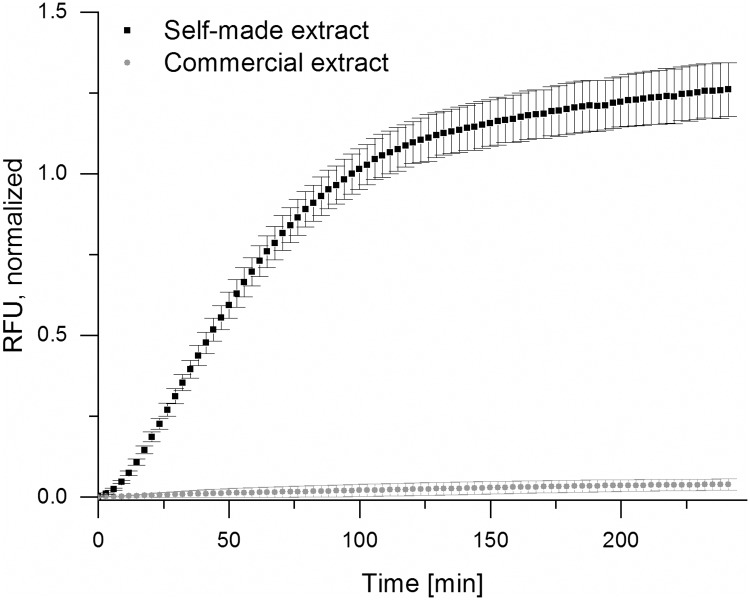
sfGFP production on paper (C350L) with self-made cell extract compared to sfGFP production on paper with a commercial extract provided by Promega. Shown are the mean values of three biological replicates ± standard deviation. Normalization was carried out as described in the Materials and Methods section.

The self-made extract resulted in a significantly higher yield of sfGFP compared to a commercial *E*. *coli* extract (Promega), demonstrating the suitability of the self-made extract for paper-based applications (Fig 4). The activity of the commercial extract seemed to be somewhat lower compared to previous reported findings of Pardee et. al. [18], the reasons for this might be batch-to-batch variation, the paper type used, or other aspects of our specific setup. When the paper discs were lyophilized directly after the addition of the reaction mix, they could be stored at room temperature for at least six days. The addition of water initiated the cell-free reaction and resulted in sfGFP production (S5 Fig). To avoid possible detrimental effects of humidity on the lyophilized reaction, the tubes with the paper discs inside were sealed, which improved the storability. Nevertheless, the fluorescence intensity was about ten times lower than during CFPS in solution, indicating that further optimization of the storage conditions should be considered.

The optimized reaction conditions were subsequently employed for the construction of an extensible, cell-free biosensor.

#### Detection of the WHO safety limit for mercury in drinking water using CFPS

**Fig 5:**
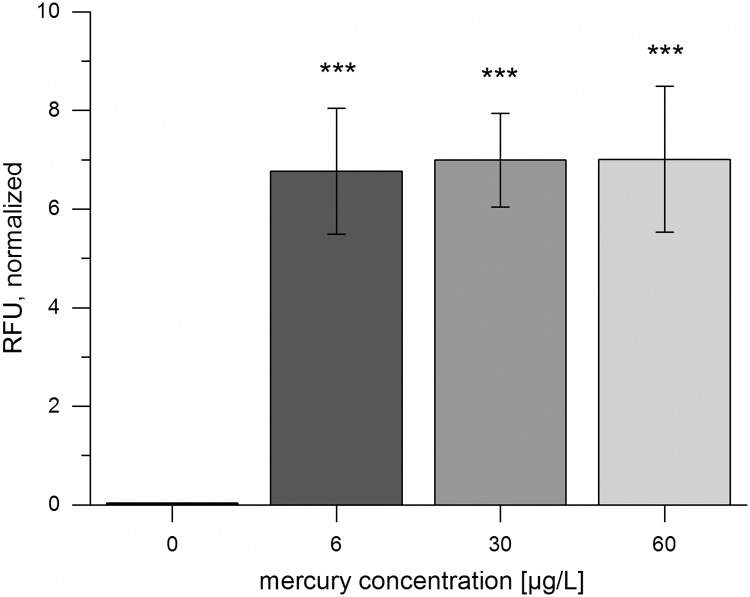
Cell-free detection of mercury in solution. Expression of sfGFP in a CFPS reaction with 10 nM of the reporter construct BBa_K1758344 and a cell extract containing MerR at various concentrations of Hg(II). Fluorescence intensities normalized to cell lysate with sfGFP 60 min after reaction initiations are shown. A cell extract prepared from *E*. *coli* KRX harboring BBa_K1758340 was used. Error bars represent the standard deviation of four biological replicates. The same cell extract batch was used for all experiments.

A modified version of the mercury sensor designed by the iGEM team Peking 2010 [53] was used for the detection of mercury. This sensor system demonstrates the sensitivity of biosensors based on CFPS, as it was possible to detect a mercury concentration of 6 μg/L (Fig 5), the WHO safety limit for mercury in drinking water [1]. Paper-based testing confirmed the results of the in-solution-detection (S6 Fig).

The high sensitivity is corroborated by the observation that the concentration of 6 μg/L appears to lie within the saturation range of the biosensor, removing all of the repressor proteins from the operators. To quickly determine whether a water sample surpasses this safety threshold or not, the detection limit can be adjusted in future work by varying the composition of the CFPS reaction and the application used for user-friendly analysis (see below).

#### Development of a novel biosensor—GHB detection

We developed a biosensor for detection of γ-butyric acid (GHB) and its precursor γ-butyrolactone (GBL), often used as date rape drug. The BlcR protein from *A*. *tumefaciens* was identified as a potential candidate for a GHB detection system [13]. An *E*. *coli* KRX strain carrying the biosensor device for detection of GHB and GBL (BBa_K1758377) was first used for an *in vivo* analysis of the sensor. The GHB-sensitive BlcR protein was constitutively expressed under the control of P_T7_.

**Fig 6:**
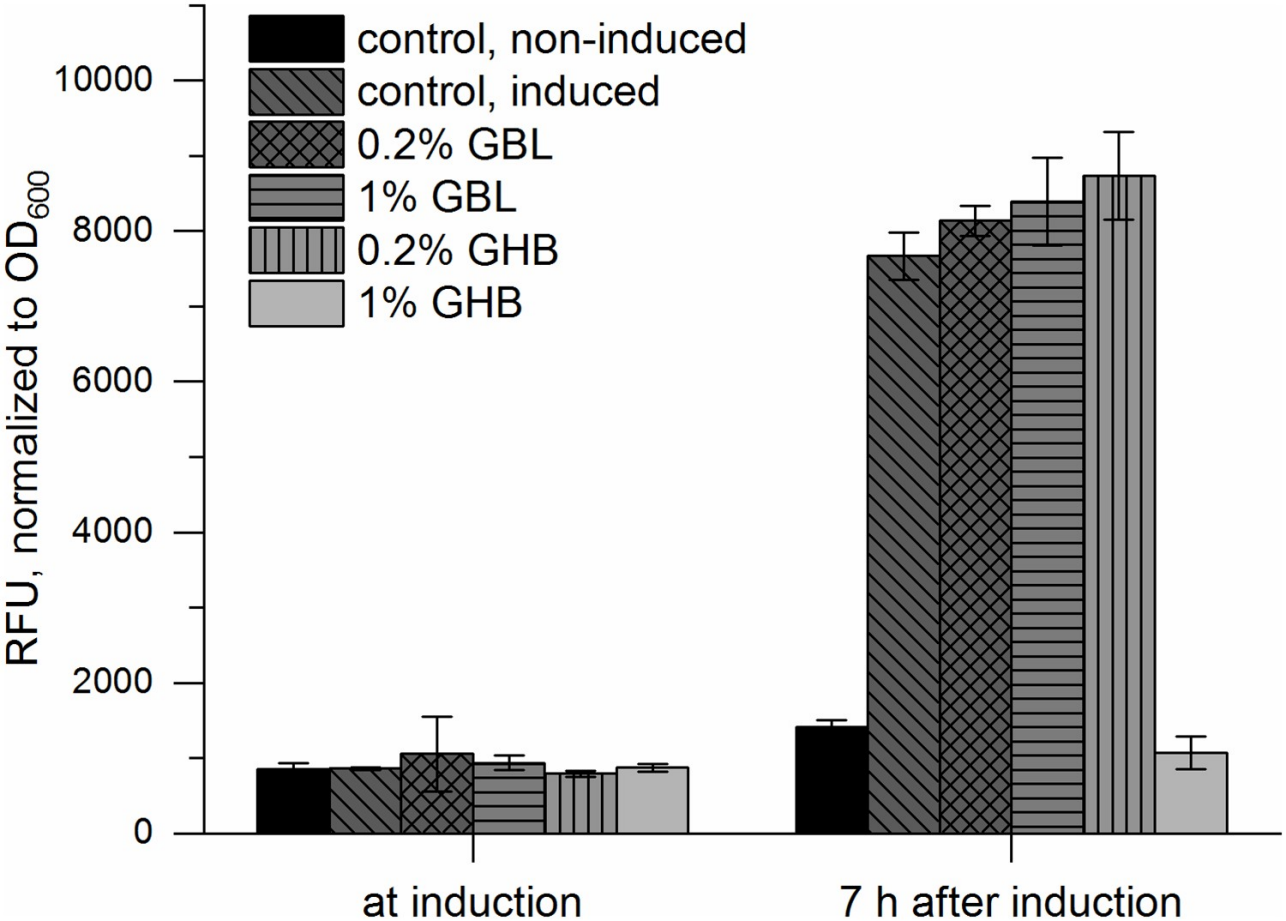
*In vivo* characterization of the GBL / GHB sensor. *E*. *coli* KRX harboring BBa_K1758377was exposed to various concentrations of GBL and GHB. All experiments were performed with biological triplicates, error bars represent the standard deviation. The fluorescence signal was normalized to OD_600_ to account for the differences in growth behavior. All samples except the non-induced control had been induced to express T7 polymerase at OD_600_ = 0.7 -0.8.

*In vivo* analysis showed a limited reaction of the BlcR repressor at 0.2% GHB. Up to 1% GBL in the reaction mix resulted in slightly increased fluorescence signals (Fig 6). This increase was not significant (p > 0.05) for any of the *in vivo* reactions. Therefore, the detection of GHB / GBL in water or beverages by an *E*. *coli* whole-cell biosensor based on BBa_K1758377 is not feasible. Furthermore, supplementation of 1% GHB resulted in growth inhibition of *E*. *coli*, which may have entailed low sfGFP expression and therefore a low fluorescence signal.

The potentially toxic analytes GHB and GBL suppressed the expression of sfGFP in solution based CFPS without BlcR. Amounts of 0.3% of both compounds were sufficient to significantly inhibit protein synthesis (S7 Fig).

**Fig 7:**
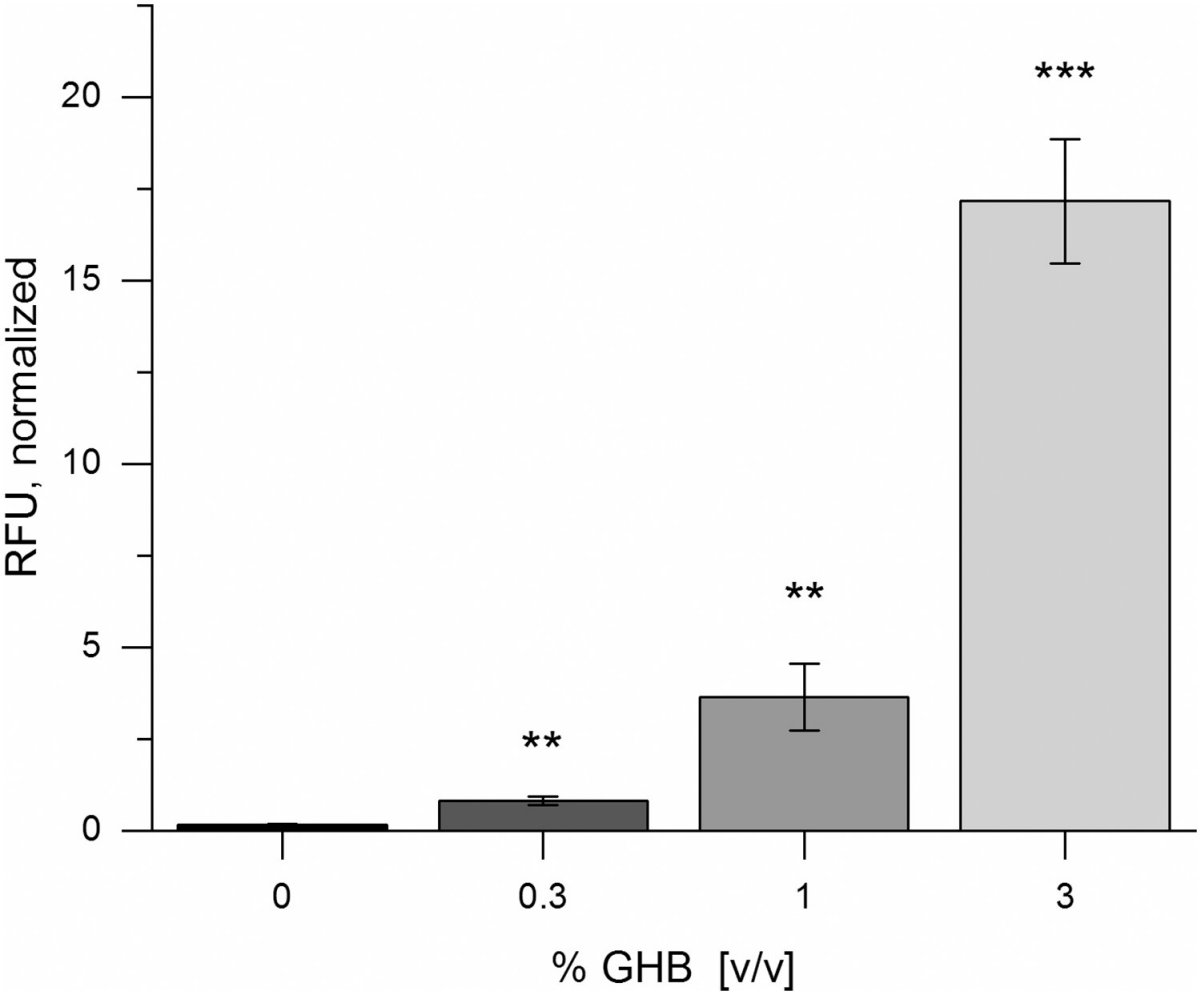
Detection of GHB in solution using cell extract containing BlcR. Shown are the mean values ± standard deviation of four biological replicates. **: p < 0.01; ***: p < 0.001. 10 nM of BBa_K1758376 were used as DNA template. Fluorescence signals were first normalized to cell lysate with sfGFP, then normalized to sfGFP expression in standard CFPS in the presence of corresponding amounts of GHB (S7 Fig). The same cell extract batch was used for all experiments.

*In vitro* experiments conducted with extract of an *E*. *coli* KRX strain that pre-expressed BlcR (BBa_K1758370) showed a reduced inhibitory effect. After normalizing the fluorescence intensity to the in-solution-CFPS sfGFP expression in the presence of corresponding amounts of GHB, an increase of fluorescence alongside the increased amount of analyte could be observed (Fig 7). Consequently, GHB detection with a cell-free system was shown to be feasible. Performing the reaction on paper discs confirmed this result, however, the signal-to-noise ratio was lower compared to the mercury on-paper biosensor (S6 and S8 Figs). Potentially, supplementation of purified BlcR as well as optimization of the overall extract activity by means of tailor-made extract preparation will enhance the performance of the biosensor. Moreover, this could possibly lead to direct GHB detection without the need for normalizing the fluorescence signal to account for the detrimental effects of the substance on CFPS. Nevertheless, this is the first time a functional biosensor for the detection of the date rape drug GHB has been constructed. Importantly, the *in vitro* approach was superior to a whole-cell biosensor system for this application (Figs 6 and 7).

### Simple and affordable output signal processing

While sfGFP is the ideal reporter protein for *in vitro* protein synthesis, small amounts are not visible to the human eye [35]. Therefore, a two-filter system suitable for use with a smartphone was developed. One light filter was placed in front of the flash (excitation) and the other one in front of the camera (emission). Different filter combinations were tested to improve the signal to noise ratio. The filter Tokyo Blue (#071 in the Lee color filter catalogue, maximum transmission at 445 nm [41]) in front of the flash combined with Twickenham Green (#736, maximum transmission at 525 nm [41]) in front of the camera generated the lowest background signal. This approach can be applied to other fluorescent proteins by employing different light filter combinations. For example, monomeric red fluorescent protein (mRFP) can be detected by combining Twickenham Green to excite the fluorophore and Light Red (#182, maximum transmission at 690 nm [41]) to photograph the emitted light (S9 Fig).

**Fig 8:**
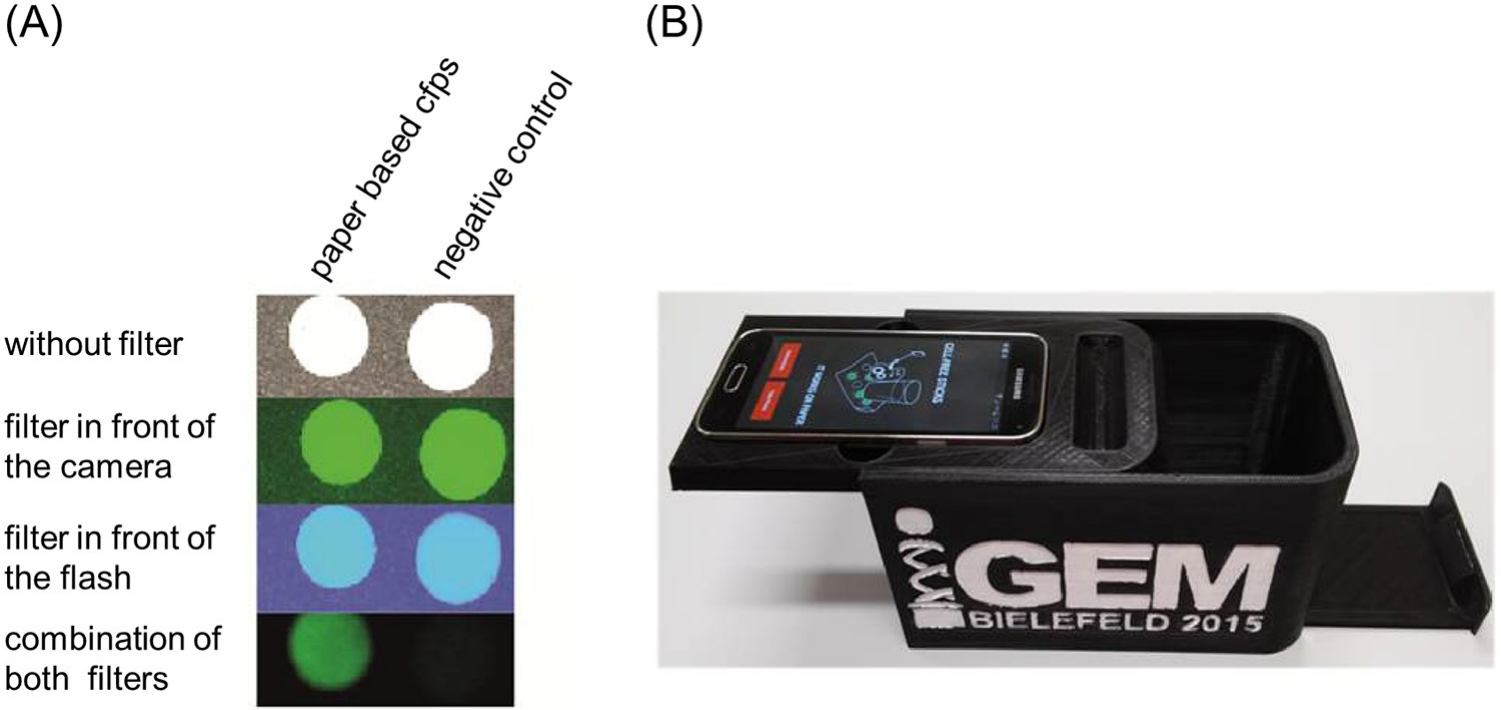
Fluorescence readout via an appropriate filter combination, a black case and a smartphone. A: Images of paper based CFPS and the corresponding negative control with different light filters. Only with both filters, the green fluorescence of sfGFP can be distinguished from the background. B: 3D-printed case for biosensor analysis.

It was not possible to detect the fluorescence signal of a CFPS positive control without any or with only one filter (Fig 8A). Only the combination of two filters allowed differentiating between the positive and the negative control. Exact positioning of the filters in front of the camera and the flash is important in order to generate pictures appropriate for detection. In addition, a dark environment is necessary for high quality images. Therefore, a black case was designed and produced by 3D printing (S1 File). The top smartphone inlay can be specifically adapted to different smartphone types. The paper discs can be placed on the push loading drawer and be inserted into the box (Fig 8B).

In order to help the user, distinguish real signals from background noise and facilitate the interpretation of the results, a smartphone application was programmed (S2 File). The 3D-printed black case ensures the correct location of the filters and reproducible conditions in terms of distance and darkness for the imaging process. With the help of this application fixed points on the images can be chosen and the median green value of the paper discs can be analyzed. Considering control reactions, the app calculates whether a contamination is present. As a higher number of paper discs can be measured in parallel, the output is a list of the analytes in the sample. In addition, the application provides background information about different heavy metals as well as GBL and their potential health risks.

## Summary and outlook

Homemade *E*. *coli* cell extract was established for paper-based applications. Cell-free protein synthesis of the reporter protein sfGFP worked well on paper when homemade extract was employed. The design of the genetic template was found to be of high importance to minimize transcription and translation limitations. The *in vitro* protein synthesis platform can be readily used for many applications, including synthetic gene networks [54], synthesis of proteins with non-natural amino acids [22], screening of enzymes [55] and others.

In this project, CFPS enabled the construction of a cell-free, paper-based biosensor for water quality assessment. The ion Hg(II) and the small molecule GHB, both hazardous for humans, served as analytes and were detected by employing MerR and BlcR, respectively, as transcriptional regulators in the cell-free reaction. Mathematical modeling of biosensor behavior demonstrated that pre-expressing a repressor protein is more favorable than expressing it in tandem with the reporter protein. The proposed model builds a strong basis for further optimization of biosensor systems based on CFPS and aids the understanding of the underlying mechanisms [24,25].

Two mechanisms may lead to successful detection of the analytes via fluorescence levels: Repressor and activator proteins may act as amplifiers of reporter production upon induction with their respective target analyte. Furthermore, detrimental effects of the analytes on CFPS may be decreased when MerR or BlcR are present from the beginning, as they bind the analytes, the consequence being that these cannot act on the transcription or translation machinery. The relationship of these mechanisms as well as the impact of the analyte should be addressed in further studies

The GHB-binding repressor protein BlcR was employed as a novel biosensor element. Our data suggest that a BlcR-based GHB / GBL sensor is not suited to be used as a whole cell *E*. *coli* biosensor, but amenable for *in vitro* applications. Identification of GHB in drinking water is feasible with the presented system. Nevertheless, optimization is needed to increase sensitivity and signal-to-noise ratio.

Key challenges for the construction of applicable paper-based CFPS biosensors are response time, robustness, specificity and storability. The response time of the proposed biosensor is relatively fast with approximately one hour. For quantification, control reactions are important to account for the effects analytes have on CFPS. In this regard, manipulation of either the concentration of transcription factor or its corresponding operator site may lead to fine-tuning of sfGFP expression levels, thereby enabling precise quantification of the analyte. Optimization of extract preparation and activity of both MerR and BlcR extracts may readily lead to a higher signal-to-noise ratio. Nevertheless, varying quality of cell extract batches, as often described in the literature [54], must not be overlooked. Furthermore, possible cross-reactions in water samples with multiple analytes have to be critically investigated in future experiments. However, mercury biosensors based on the same biological elements have been found to be highly specific in previous studies [56, 57]. Moreover, as biosensors with different specificities can easily be used in parallel in our proposed system, cross-reactivities of individual sensing elements could potentially be compensated for.

Cell-free production of sfGFP is possible at 25 °C and potentially even at lower temperatures. Thus, an application in the field is quite conceivable. The most favorable storage conditions for the freeze-dried biosensor remain to be investigated. In particular for field usage, storability and handling have to be considered. Pardee and colleagues reported a shelf life of lyophilized extract of one year at room temperature, at least under conditions of light protection, inert gas atmosphere and presence of silica gel desiccation packages [18].

With the herein developed smartphone-based setup for the detection of fluorescence, a cheap and easy method is proposed that enables the use of fluorescent reporter proteins in assay applications in the open field. This is a valuable alternative to colorimetric outputs, for example via beta-galactosidase [18]. To ensure a more accurate quantification of the analytes, a reference curve could be produced with each measurement. Another improvement would be to implement a more dynamic recognition script for the paper disc spots.

Cell-free tools have a great advantage over whole-cell biosensors regarding biosafety, especially when combined with lyophilization [58]. Cell-free and paper-based biosensors hold great potential for the detection of various substances in regions where analytical laboratories are rare. With the possibility of identifying new allosteric transcription factors [59], simple detection of more and more substances is within reach.

## Supporting information

S1 Text Design of an efficient 5’-UTR for *in vitro* transcription. (PDF)

S1 Table Composition of a standard cell-free reaction. Unless otherwise noted, 10 nM of mini-prepped plasmid were used as DNA template for experiments.
(XLSX)

S2 Table Overview of species used in the biosensor model. (XLSX)

S3 Table Overview of parameters used in the biosensor model. (XLSX)

S1 Appendix Differential equations for Biosensor Model calculation. (PDF)

S1 Fig Temperature sensitivity of CFPS reactions. Shown are relative fluorescence units (RFU) of positive control setups (10 nM P_T7_-UTR-sfGFP, BBa_K1758102) in solution (15 μL) at various temperatures after 60 and 150 min, respectively. For each temperature test, a new reaction was prepared as the plate reader could only generate one temperature at a time. Measurement specifications were identical in every run, as depicted in the Materials and Methods section, with a manual gain of 70. Error bars represent the standard deviation of four biological replicates.
(TIF)

S2 Fig Effect of *E*. *coli* RNase E inhibitor RraA on CFPS reactions. The increase of relative fluorescence units (RFU) normalized to cell lysate with sfGFP over time is shown. RraA in 50 mM Hepes buffer, pH 7.2 was added at a final concentration of 0.3 mg/mL (black squares). In control reactions, the same volume of 50 mM HEPES buffer, pH 7.2 (dark grey dots) and water (light grey triangles), respectively, was used. The normalized fluorescence signal in the RraA supplemented reaction is significantly higher (p < 0.05) than the HEPES buffer supplemented reaction after 70 min. For each reaction, biological triplicates were measured (error bars represent the standard deviation). 10 nM of P_T7_-UTR-sfGFP (BBa_K1758102) DNA template were used for each reaction.
(TIF)

S3 Fig Comparison of experimental results and model predictions. (A) Experimental data for sfGFP expression at various plasmid concentrations (squares, with error bars showing the standard deviation of three biological replicates) was used as training data for the model. The solid lines represent the model results after data fitting. (B) Validation using data for two plasmid concentrations that had not been part of the training data set. Solid lines represent predictions by the model, squares with error bars show the standard deviation of three biological replicates. (C) Competition for resources as predicted by the model (solid lines) and as observed in experiments (squares, with error bars showing the standard deviation of three biological replicates). The sfGFP fluorescence was measured without a second plasmid and with an equimolar amount of mRFP1 plasmid. As predicted by the model, the addition of a second plasmid resulted in a decrease in sfGFP production. This decrease was slightly lower than predicted, which might indicate that mRFP1 was not expressed as well as sfGFP.
(TIF)

S4 Fig Comparison of biosensor designs using model predictions. (A) Influence of the concentrations of reporter and repressor plasmid when a co-expression of the repressor is desired. For each plasmid ratio, sfGFP expression was simulated for analyte concentrations spanning six orders of magnitude in order to give impression of the dynamic range. The resulting sfGFP concentrations are represented by lines with identical formatting. (B) Comparison of pre-expression and co-expression of the repressor. Pre-expression leads to a lower background signal and a higher signal intensity in the presence of an analyte. To simulate co-expression, equimolar amounts (8 nM) of reporter and repressor genes were assumed, while pre-expression was simulated assuming 8 nM reporter plasmid and 300 nM repressor dimer.
(TIF)

S5 Fig Storability of lyophilized on-paper cell-free reactions. Shown are fluorescence units (RFU) of positive control setups (10 nM P_T7_-UTR-sfGFP, BBa_K1758102) on paper discs (Munktell C350L) normalized to cell lysate with sfGFP over time. After lyophilization of the freshly prepared cell-free reactions on paper discs, the latter were stored for six days at room temperature in closed 1.5 mL reaction tubes. Some of the tubes were sealed with adhesive film (black squares) directly after lyophilization to avoid possible detrimental effects on the lyophilized reaction caused by humidity. Afterwards, 15 μL water were added to the discs to initiate the CFPS reaction. Fluorescence was monitored in a plate reader (see Materials and methods section).
(TIF)

S6 Fig Detection of Hg(II) with CFPS on paper (C350L). Shown are the relative fluorescence units (RFU) of cell-free reactions supplemented with no or 6 μg/L Hg(II), normalized to cell lysate with sfGFP, 60 and 150 min after reaction initiation, respectively. Error bars represent the standard deviation of biological triplicates. ***: p < 0.001.
(TIF)

S7 Fig Inhibition of in-solution *E*. *coli* ER2566 CFPS by γ-hydroxybutyrate (GHB) and γ-butyrolactone (GBL). Shown are the relative fluorescence units (RFU) normalized to cell lysate with sfGFP for various percentages of GHB and GBL, respectively, 60 min after reaction start. 10 nM P_T7_-UTR-sfGFP (BBa_K1758102) was used as DNA template. Both substances strongly inhibit standard CFPS, with GHB having a more detrimental effect at concentrations above 0.3% (v/v). Error bars represent the standard deviation of four biological replicates.
(TIF)

S8 Fig Detection of GHB with CFPS on paper (C350L). Shown are the relative fluorescence units (RFU) of cell-free reactions supplemented with 0%, 1% or 2% GHB 60 and 150 min after reaction initiation, respectively. The fluorescence signals were first normalized to cell lysate with sfGFP as described in the Materials and Methods section, and then normalized to sfGFP expression in paper-based CFPS without BlcR in the presence of corresponding amounts of GHB. Error bars represent the standard deviation of four biological replicates, calculated using Gaussian error propagation.
(TIF)

S9 Fig mRFP fluorescence measurement with two-filter combination. Shown are three photographs of the same four reaction tubes to test different filter combinations to detect mRFP fluorescence. The tubes containing nothing, lysis buffer, cell lysate of *E*. *coli* KRX culture harboring BBa_K1758106 that was not induced to express mRFP, and cell lysate of a culture from the same strain that was induced to express mRFP. No filters were used to photograph the tubes in the top row. The picture in the middle was taken with Light Red in front of the camera and Dark Yellow Green in front of the flash. The bottom photo was taken with the optimal filter combination Twickenham Green in front of the flash and Light Red in front of the camera.
(TIF)

S1 File 3D printing template for black case. (ZIP)

S2 File Smartphone application for biosensor result interpretation. (ZIP)

S1 Dataset Excel sheet with raw data. (XLSX)
