# Supplementary material for: A paper-based, cell-free biosensor system for the detection of heavy metals and date rape drugs
Source: PLoS One. 2019 Mar 6;14(3):e0210940. doi: 10.1371/journal.pone.0210940 (PMC6402643; doi:10.1371/journal.pone.0210940)
Supplement: S1 Text — (PDF) [file pone.0210940.s001.pdf]

S1 Text: Design of an efficient 5'-UTR for *in vitro* transcription

The 5'-untranslated region (5'-UTR), is of major importance for an efficient reaction. We designed a new translation enhancing 5'-UTR (BBa_K1758100) based on the literature as depicted below and inserted it into P_{T7}-sfGFP (BBa_I746909) to create P_{T7}-UTR-sfGFP (BBa_K1758102)

5'- AATAATTTTGTTTTAACTTTAAAAAAAAAAAAAGAAGGAGAATAATCT - **ATG** - 3'

The T7 promoter is located directly upstream and the start codon ATG is located downstream of this sequence. In the control plasmid with BBa_I746909, the following sequence is used between the promoter and the start codon:

5' - TACTAGAGAAAGAGGAGAAATACTAG – **ATG - 3'**

These are the features of the optimized 5'UTR sequence:

- AATAATTTTGTTTTAACTTTAA
    - To increase the efficiency of translation initiation, the T7 *g10* leader sequence can be employed. This sequence contains the so called epsilon motif TTAACTTTA [58], and was originally described by Olins *et al*. [32]. It enhances the binding of the mRNA to the 16 S rRNA [32, 33, 41], and Olins *et al*. showed that this sequence can massively improve heterologous gene expression in *E. coli* [32].
- poly-A-spacer
    - With kinetic studies, Takahashi *et al*. showed that a spacer between the epsilon motive and the RBS improves the translation rate *in vitro*. This

works unless the spacer interacts with the 30S subunit of the ribosome, which is not the case for example for an all-adenine spacer [33]. They further determined a 10-A-spacer as suitable when using *E. coli* S30 extracts.

- GAAGGAG
  - The effect of the ribosome binding site (RBS) on *in vitro* translation has been investigated extensively [33 - 35]. Most important for an efficient reaction is a defined distance of 4-9 bases of the RBS to the following start codon [34, 35]. The strength of the RBS has a minor effect as long as it is not too weak [33, 34].
- AATAATCT
  - According to Lentini *et al*., the sequence composition between RBS and start codon affects the expression level of the following gene. An AT-rich region gives the best results, whereas expression is lower with the biobrick scar TACTAGAG for example [35].
