# Supplementary material for: A paper-based, cell-free biosensor system for the detection of heavy metals and date rape drugs
Source: PLoS One. 2019 Mar 6;14(3):e0210940. doi: 10.1371/journal.pone.0210940 (PMC6402643; doi:10.1371/journal.pone.0210940)
Supplement: S1 Appendix — (PDF) [file pone.0210940.s005.pdf]

S1 Appendix: Biosensor Model.

The differential equations are the following:

$$\frac{d[m_R]}{dt} = v_{TX2} \cdot \frac{[G_R]^2}{{K_{TX2}}^2 + [G_R]^2} - \lambda_{m2} \cdot [m_R]$$

$$\frac{d[R]}{dt} = k_{TL2} \cdot [TLR] \cdot \frac{[m_R]^3}{{K_{TL2}}^3 + [m_R]^3 + [m_F]^3} - 2 \cdot k_{2R} \cdot [R]^2 + 2 \cdot k_{-2R} \cdot [R_2]$$

$$\frac{d[R_2]}{dt} = k_{2R} \cdot [R]^2 - k_{-2R} \cdot [R_2] - k_r \cdot [R_2] \cdot [O] + k_{-r} \cdot [R_2O] - k_{dr1} \cdot [A]^2 \cdot [R_2] + k_{-dr1} \cdot [R_2A_2]$$

$$\frac{d[R_2O]}{dt} = k_r \cdot [R_2] \cdot [O] - k_{-r} \cdot [R_2O] - k_{dr2} \cdot [A]^2 \cdot [R_2O] + k_{-dr2} \cdot [R_2A_2] \cdot [O]$$

$$\frac{d[A]}{dt} = -2 \cdot k_{dr1} \cdot [A]^2 \cdot [R_2] + 2 \cdot k_{-dr1} \cdot [R_2A_2] - 2 \cdot k_{dr2} \cdot [A]^2 \cdot [R_2O] + k_{-dr2} \cdot [R_2A_2] \cdot [O]$$

$$\frac{d[R_2A_2]}{dt} = k_{dr1} \cdot [A]^2 \cdot [R_2] - k_{-dr1} \cdot [R_2A_2] + k_{dr2} \cdot [A]^2 \cdot [R_2O] - k_{-dr2} \cdot [R_2A_2] \cdot [O]$$

$$\frac{d[O]}{dt} = -k_r \cdot [R_2] \cdot [O] + k_{-r} \cdot [R_2O] + k_{dr2} \cdot [A]^2 \cdot [R_2O] - k_{-dr2} \cdot [R_2A_2] \cdot [O]$$

$$\frac{d[m_F]}{dt} = k_{TX1} \cdot \frac{[O]^2}{{K_{TX1}}^2 + [O]^2} - \lambda_{m1} \cdot [m_F] + k_{leak} \cdot [R_2O]$$

$$\frac{d[F_{\mathrm{in}}]}{dt} = k_{TL1} \cdot [TLR] \cdot \frac{[m_F]^3}{{K_{TL1}}^3 + [m_F]^3 + [m_R]^3} - k_{mat} . [F_{\mathrm{in}}]$$

$$\frac{d[F]}{dt} = k_{mat} \cdot [F_{\mathrm{in}}]$$

$$\frac{d[TLR]}{dt} = -v_{\lambda TLR} \cdot \frac{[TLR]}{K_{\lambda TLR} + [TLR]}$$
