# Supplementary figures and images for: A paper-based, cell-free biosensor system for the detection of heavy metals and date rape drugs
Source: PLoS One. 2019 Mar 6;14(3):e0210940. doi: 10.1371/journal.pone.0210940 (PMC6402643; doi:10.1371/journal.pone.0210940)

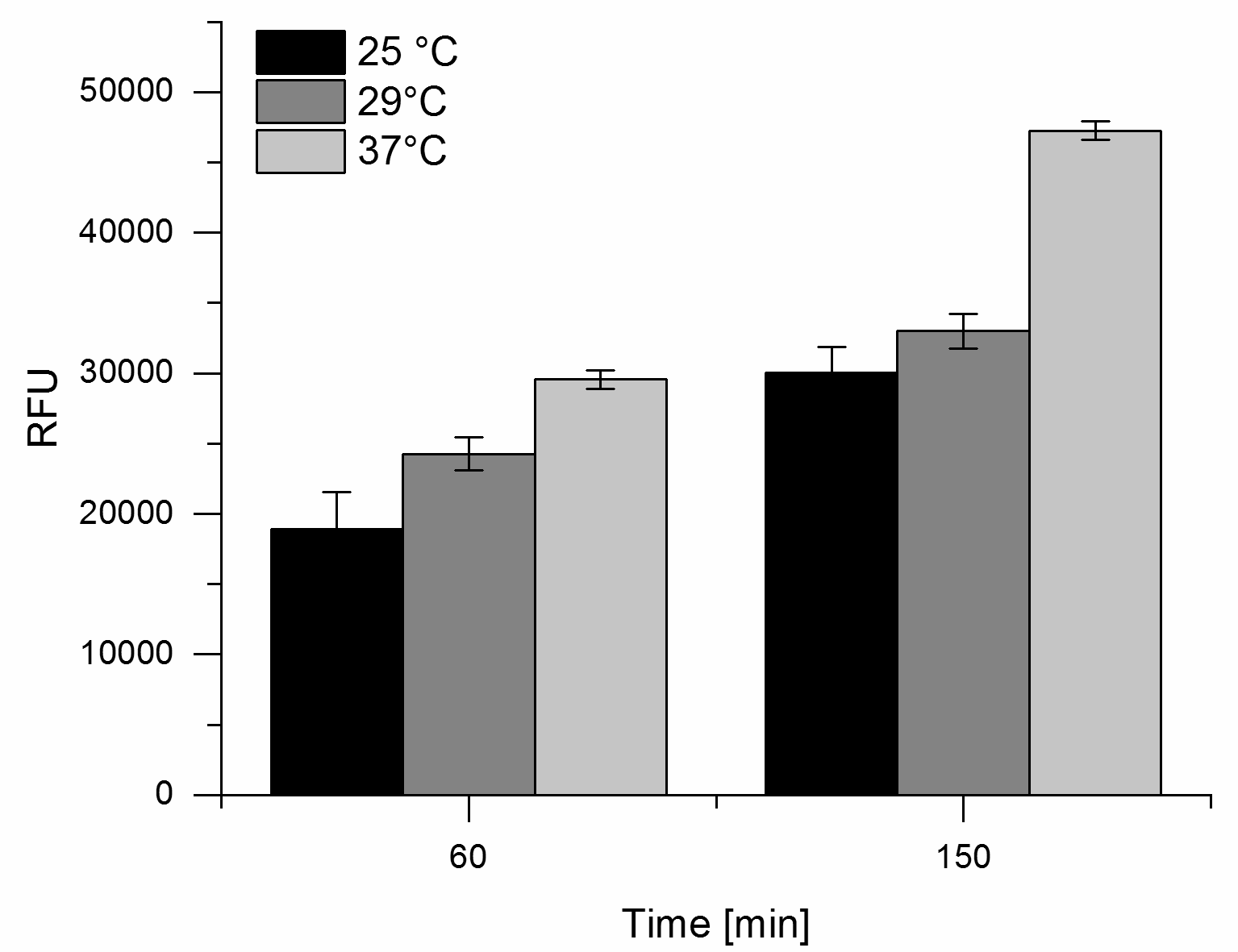

Supplement: S1 Fig — Shown are relative fluorescence units (RFU) of positive control setups (10 nM PT7-UTR-sfGFP, BBa_K1758102) in solution (15 μL) at various temperatures after 60 and 150 min, respectively. For each temperature test, a new reaction was prepared as the plate reader could only generate one temperature at a time. Measurement specifications were identical in every run, as depicted in the Materials and Methods section, with a manual gain of 70. Error bars represent the standard deviation of four biological replicates. (TIF) [file pone.0210940.s006.tif]

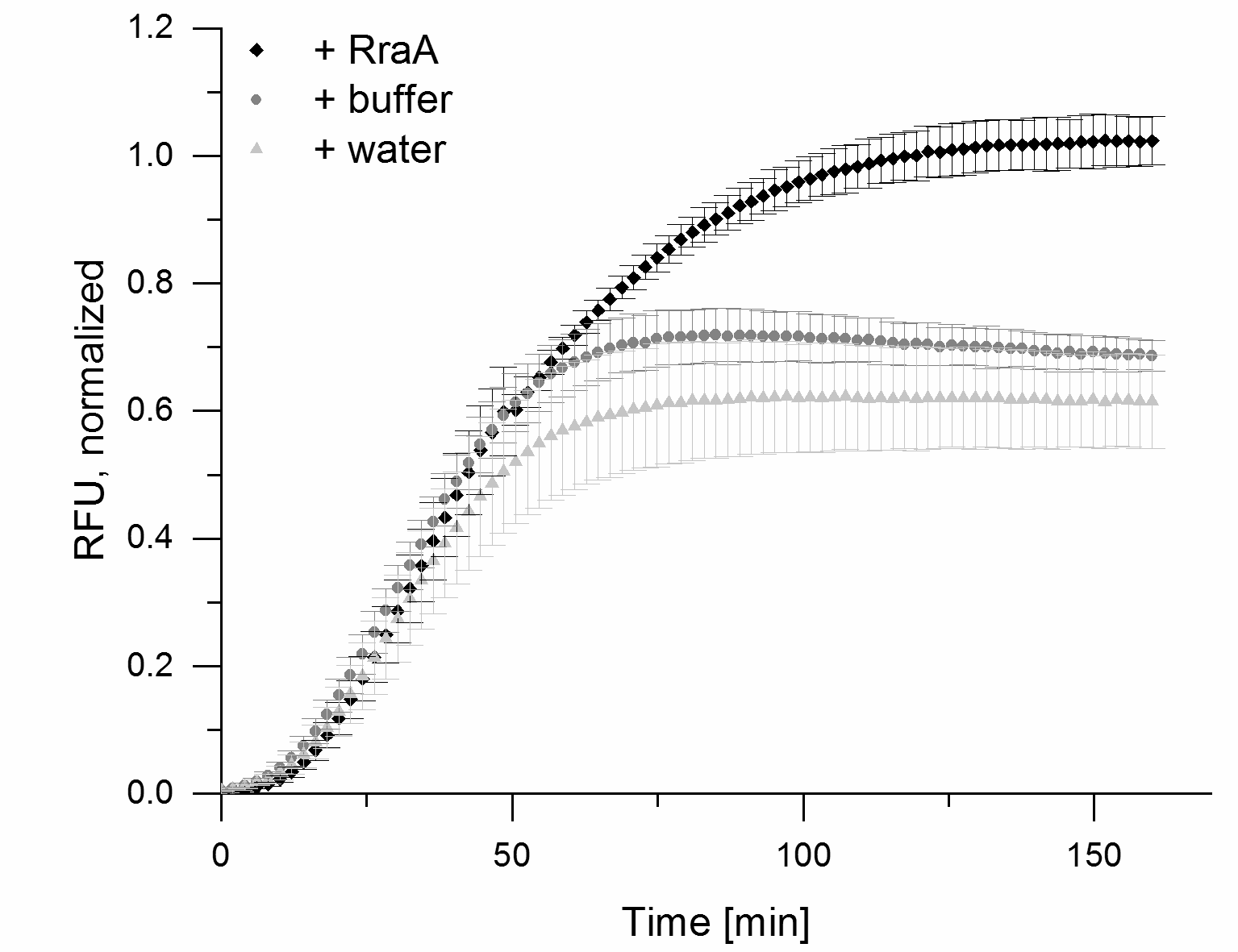

Supplement: S2 Fig — The increase of relative fluorescence units (RFU) normalized to cell lysate with sfGFP over time is shown. RraA in 50 mM Hepes buffer, pH 7.2 was added at a final concentration of 0.3 mg/mL (black squares). In control reactions, the same volume of 50 mM HEPES buffer, pH 7.2 (dark grey dots) and water (light grey triangles), respectively, was used. The normalized fluorescence signal in the RraA supplemented reaction is significantly higher (p < 0.05) than the HEPES buffer supplemented reaction after 70 min. For each reaction, biological triplicates were measured (error bars represent the standard deviation). 10 nM of PT7-UTR-sfGFP (BBa_K1758102) DNA template were used for each reaction. (TIF) [file pone.0210940.s007.tif]

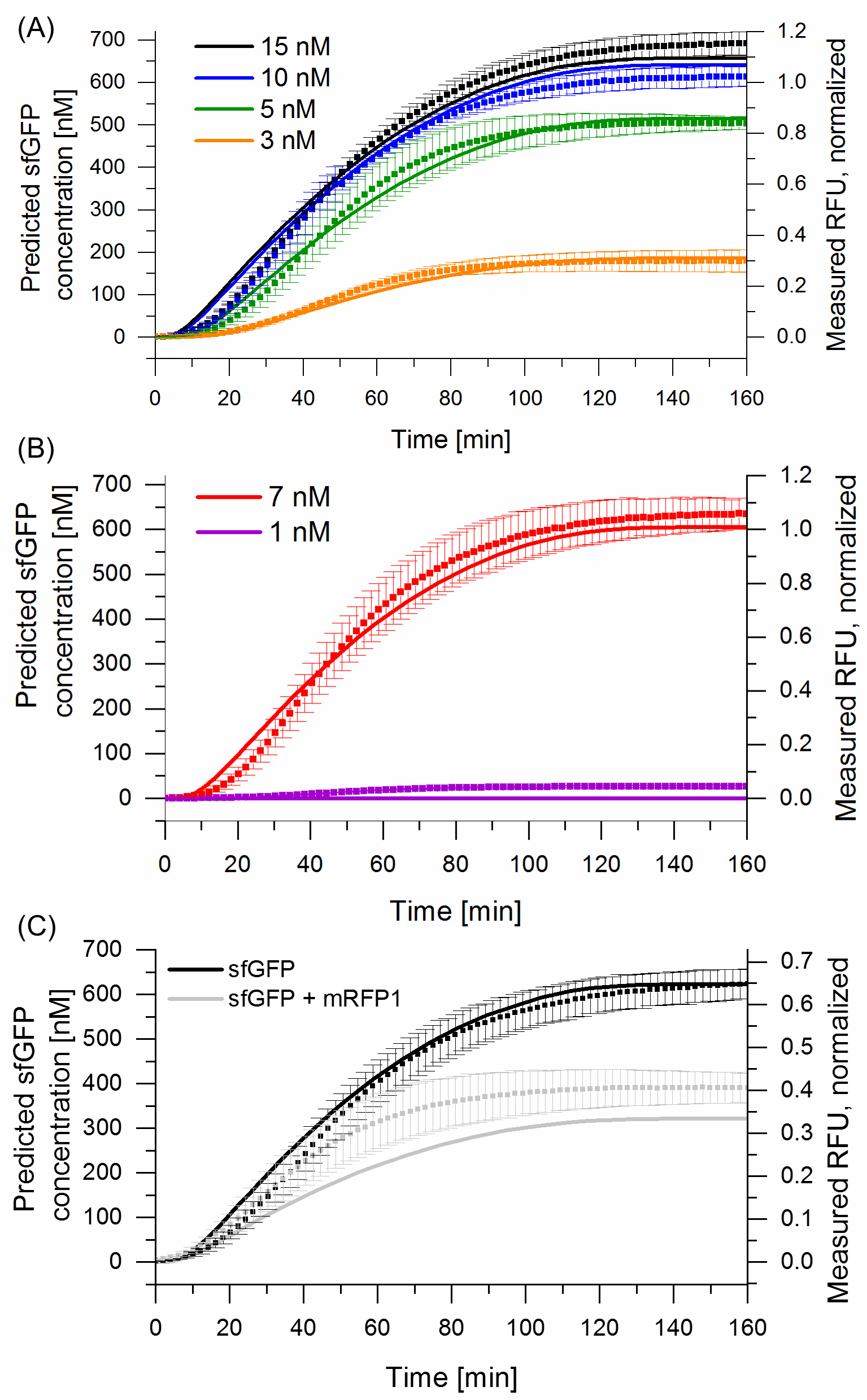

Supplement: S3 Fig — (A) Experimental data for sfGFP expression at various plasmid concentrations (squares, with error bars showing the standard deviation of three biological replicates) was used as training data for the model. The solid lines represent the model results after data fitting. (B) Validation using data for two plasmid concentrations that had not been part of the training data set. Solid lines represent predictions by the model, squares with error bars show the standard deviation of three biological replicates. (C) Competition for resources as predicted by the model (solid lines) and as observed in experiments (squares, with error bars showing the standard deviation of three biological replicates). The sfGFP fluorescence was measured without a second plasmid and with an equimolar amount of mRFP1 plasmid. As predicted by the model, the addition of a second plasmid resulted in a decrease in sfGFP production. This decrease was slightly lower than predicted, which might indicate that mRFP1 was not expressed as well as sfGFP. (TIF) [file pone.0210940.s008.tif]

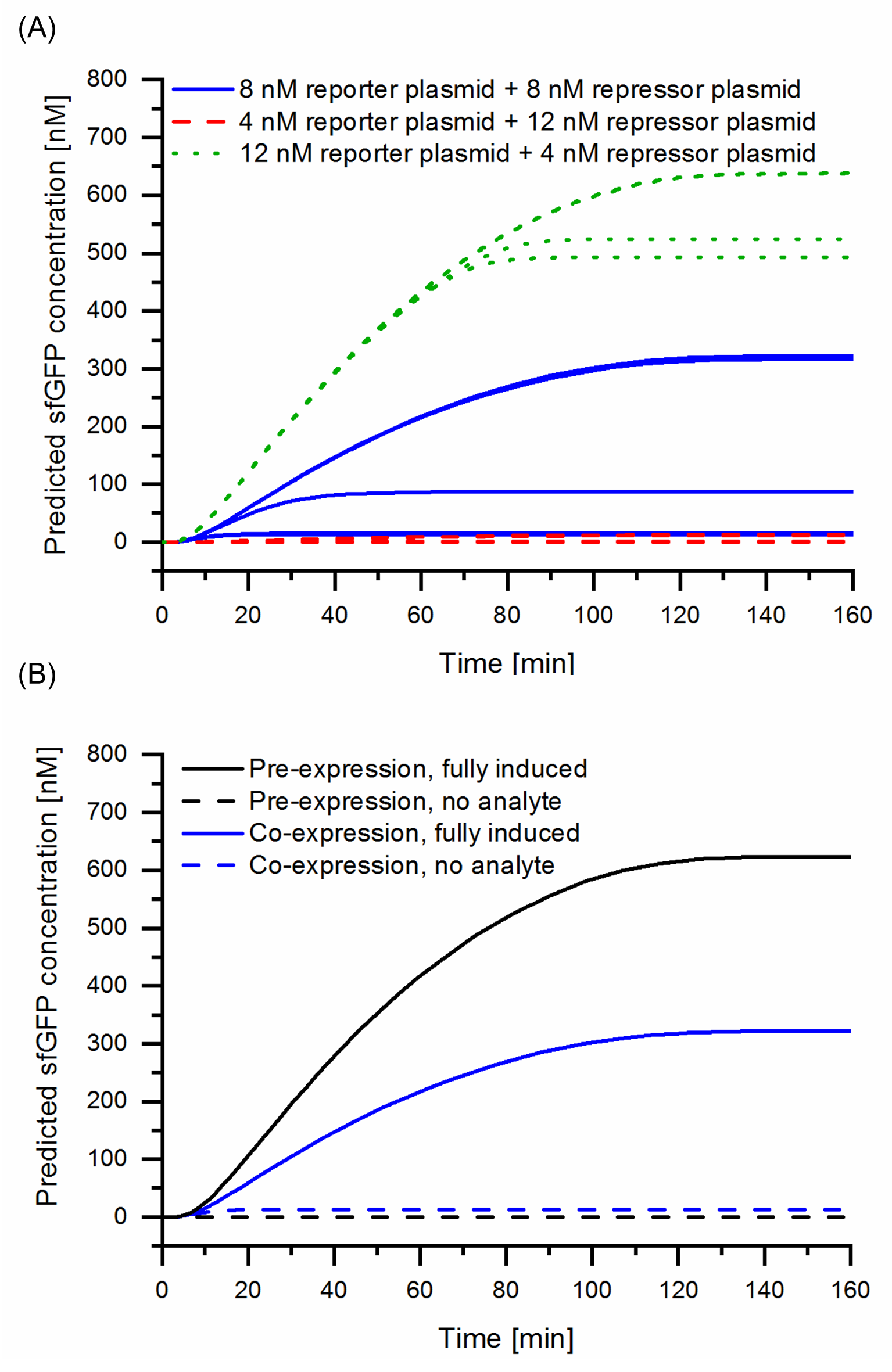

Supplement: S4 Fig — (A) Influence of the concentrations of reporter and repressor plasmid when a co-expression of the repressor is desired. For each plasmid ratio, sfGFP expression was simulated for analyte concentrations spanning six orders of magnitude in order to give impression of the dynamic range. The resulting sfGFP concentrations are represented by lines with identical formatting. (B) Comparison of pre-expression and co-expression of the repressor. Pre-expression leads to a lower background signal and a higher signal intensity in the presence of an analyte. To simulate co-expression, equimolar amounts (8 nM) of reporter and repressor genes were assumed, while pre-expression was simulated assuming 8 nM reporter plasmid and 300 nM repressor dimer. (TIF) [file pone.0210940.s009.tif]

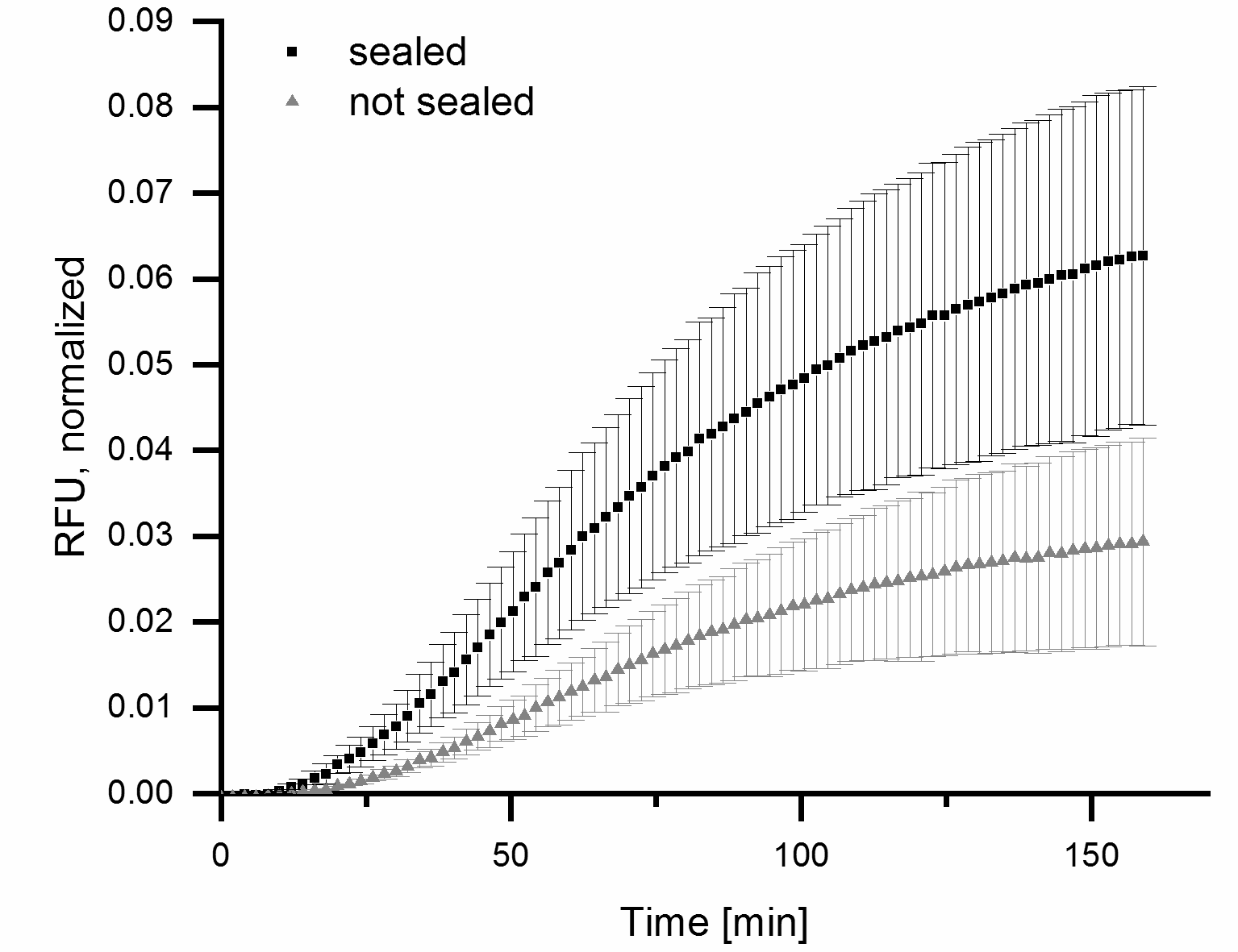

Supplement: S5 Fig — Shown are fluorescence units (RFU) of positive control setups (10 nM PT7-UTR-sfGFP, BBa_K1758102) on paper discs (Munktell C350L) normalized to cell lysate with sfGFP over time. After lyophilization of the freshly prepared cell-free reactions on paper discs, the latter were stored for six days at room temperature in closed 1.5 mL reaction tubes. Some of the tubes were sealed with adhesive film (black squares) directly after lyophilization to avoid possible detrimental effects on the lyophilized reaction caused by humidity. Afterwards, 15 μL water were added to the discs to initiate the CFPS reaction. Fluorescence was monitored in a plate reader (see Materials and methods section). (TIF) [file pone.0210940.s010.tif]

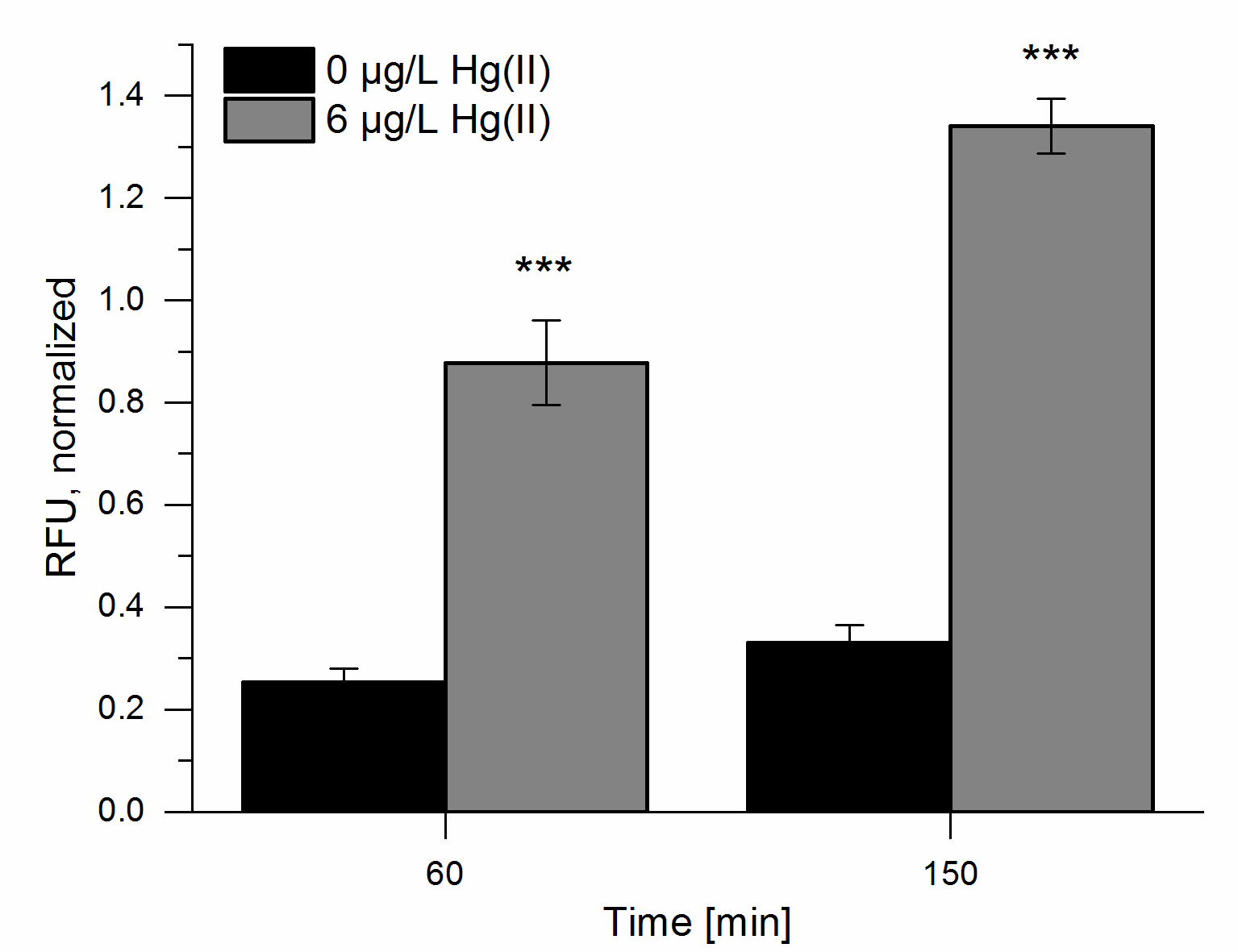

Supplement: S6 Fig — Shown are the relative fluorescence units (RFU) of cell-free reactions supplemented with no or 6 μg/L Hg(II), normalized to cell lysate with sfGFP, 60 and 150 min after reaction initiation, respectively. Error bars represent the standard deviation of biological triplicates. ***: p < 0.001. (TIF) [file pone.0210940.s011.tif]

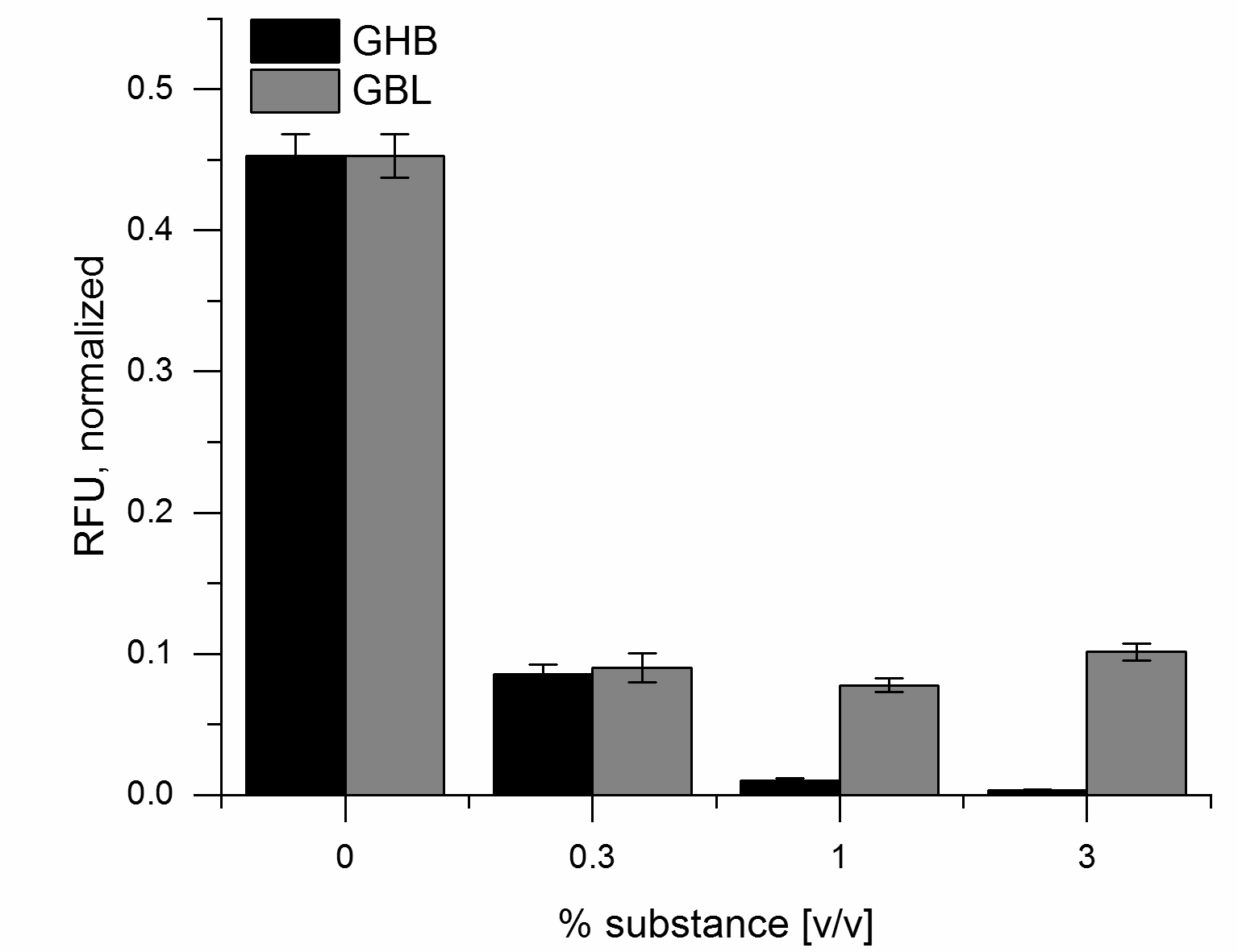

Supplement: S7 Fig — Shown are the relative fluorescence units (RFU) normalized to cell lysate with sfGFP for various percentages of GHB and GBL, respectively, 60 min after reaction start. 10 nM PT7-UTR-sfGFP (BBa_K1758102) was used as DNA template. Both substances strongly inhibit standard CFPS, with GHB having a more detrimental effect at concentrations above 0.3% (v/v). Error bars represent the standard deviation of four biological replicates. (TIF) [file pone.0210940.s012.tif]

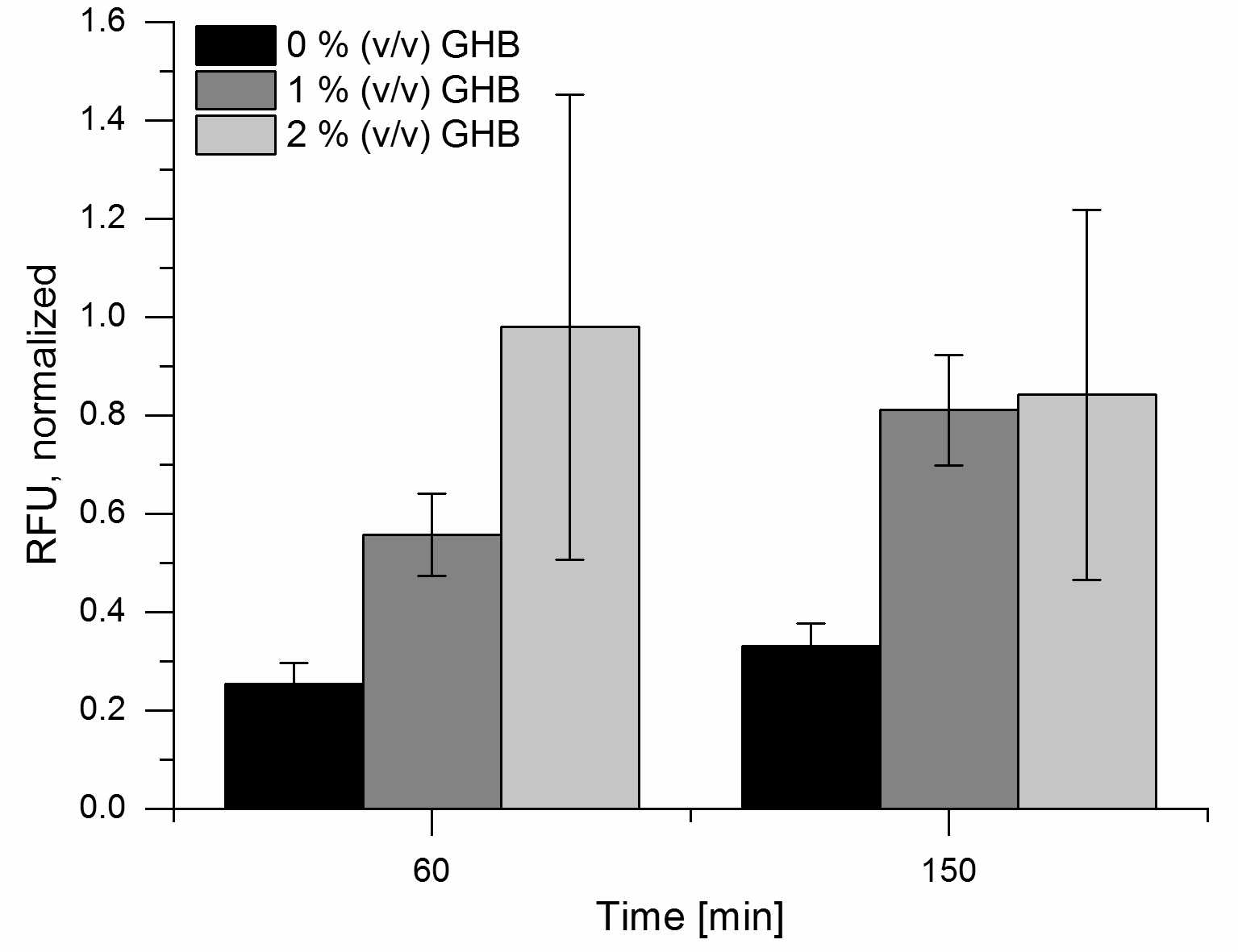

Supplement: S8 Fig — Shown are the relative fluorescence units (RFU) of cell-free reactions supplemented with 0%, 1% or 2% GHB 60 and 150 min after reaction initiation, respectively. The fluorescence signals were first normalized to cell lysate with sfGFP as described in the Materials and Methods section, and then normalized to sfGFP expression in paper-based CFPS without BlcR in the presence of corresponding amounts of GHB. Error bars represent the standard deviation of four biological replicates, calculated using Gaussian error propagation. (TIF) [file pone.0210940.s013.tif]

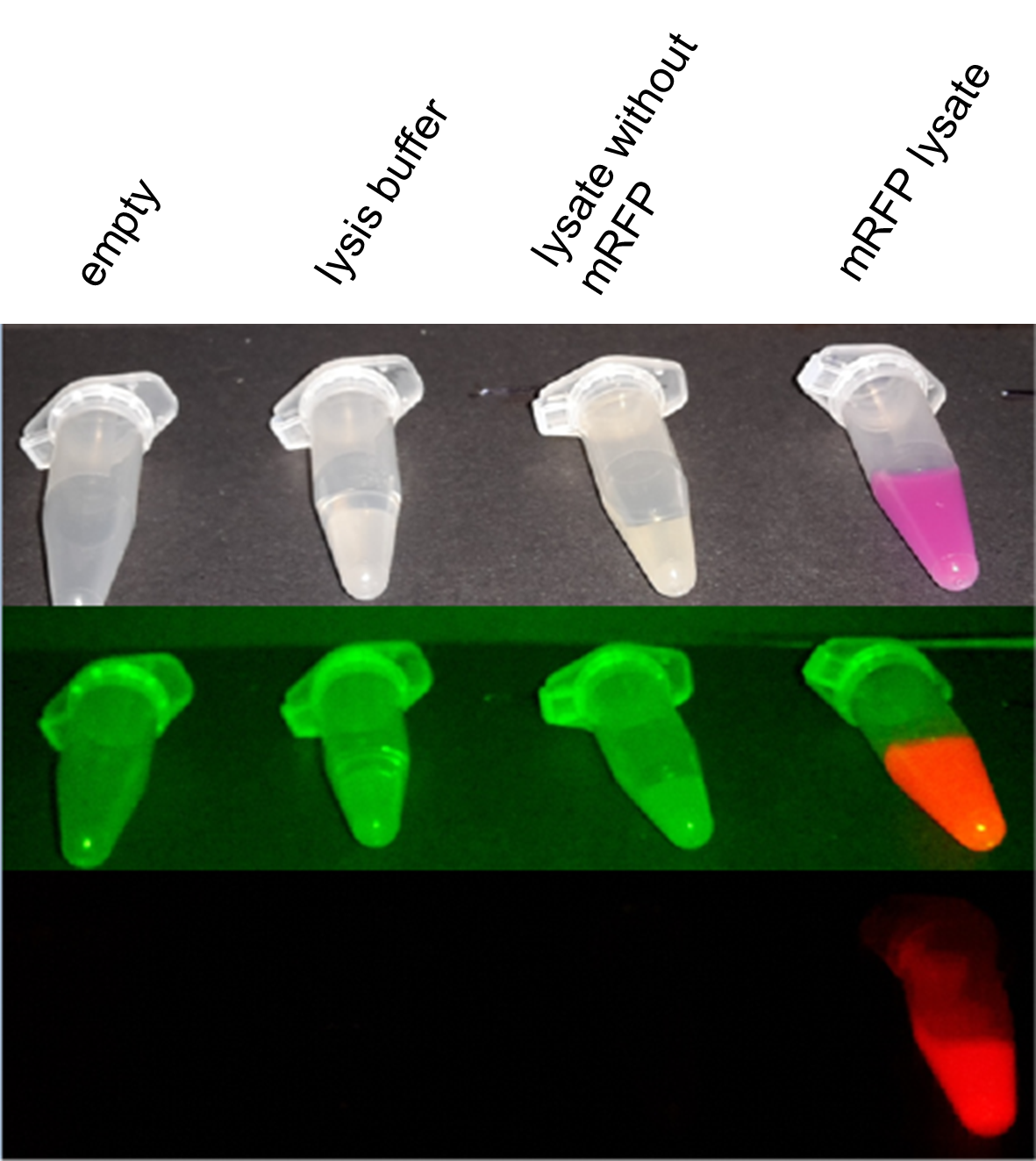

Supplement: S9 Fig — Shown are three photographs of the same four reaction tubes to test different filter combinations to detect mRFP fluorescence. The tubes containing nothing, lysis buffer, cell lysate of E. coli KRX culture harboring BBa_K1758106 that was not induced to express mRFP, and cell lysate of a culture from the same strain that was induced to express mRFP. No filters were used to photograph the tubes in the top row. The picture in the middle was taken with Light Red in front of the camera and Dark Yellow Green in front of the flash. The bottom photo was taken with the optimal filter combination Twickenham Green in front of the flash and Light Red in front of the camera. (TIF) [file pone.0210940.s014.tif]
