# Supplementary material for: A paper-based, cell-free biosensor system for the detection of heavy metals and date rape drugs
Source: PLoS One. 2019 Mar 6;14(3):e0210940. doi: 10.1371/journal.pone.0210940 (PMC6402643; doi:10.1371/journal.pone.0210940)
Supplement: S2 File — (ZIP) [file pone.0210940.s016.zip › exportToHTMLres/AndroidManifest.xml.html]

AndroidManifest.xml


|  |
| --- |
| AndroidManifest.xml |

```
<?xml version="1.0" encoding="utf-8"?> 
<manifest xmlns:android="http://schemas.android.com/apk/res/android" 
    package="de.anna.cellfreestick" > 
 
    <uses-feature 
        android:name="android.hardware.camera" 
        android:required="true" /> 
 
    <uses-permission android:name="android.permission.CAMERA" /> 
    <uses-permission android:name="android.permission.FLASHLIGHT" /> 
    <uses-permission android:name="android.permission.WRITE_EXTERNAL_STORAGE" /> 
    <uses-permission android:name="android.permission.READ_EXTERNAL_STORAGE" /> 
 
    <application 
        android:allowBackup="true" 
        android:icon="@mipmap/ic_launcher" 
        android:label="@string/app_name" 
        android:theme="@style/AppTheme" > 
        <activity 
            android:name=".MainActivity" 
            android:label="@string/app_name" > 
            <intent-filter> 
                <action android:name="android.intent.action.MAIN" /> 
 
                <category android:name="android.intent.category.LAUNCHER" /> 
            </intent-filter> 
        </activity> 
        <activity 
            android:name=".Instructions" 
            android:logo="@drawable/icon_igem" 
            android:label="@string/title_activity_instructions2" > 
        </activity> 
        <activity 
            android:name=".TakePhoto" 
            android:label="@string/title_activity_take_photo" > 
        </activity> 
        <activity 
            android:name=".Results" 
            android:label="@string/title_activity_results" > 
        </activity> 
        <activity 
            android:name=".Analysis" 
            android:label="@string/title_activity_analysis" > 
        </activity> 
        <activity 
            android:name=".HeavyMetalsDetailsActivity" 
            android:label="@string/title_activity_heavy_metals_details" > 
        </activity> 
        <activity 
            android:name=".ContaminationList" 
            android:label="@string/title_activity_contamination_list" > 
        </activity> 
    </application> 
 
</manifest>
```
