# Supplementary material for: A paper-based, cell-free biosensor system for the detection of heavy metals and date rape drugs
Source: PLoS One. 2019 Mar 6;14(3):e0210940. doi: 10.1371/journal.pone.0210940 (PMC6402643; doi:10.1371/journal.pone.0210940)
Supplement: S2 File — (ZIP) [file pone.0210940.s016.zip › exportToHTMLres/de/anna/cellfreestick/Analysis.java.html]

Analysis.java


|  |
| --- |
| Analysis.java |

```
package de.anna.cellfreestick; 
 
import android.support.v7.app.ActionBarActivity; 
import android.os.Bundle; 
import android.view.Menu; 
import android.view.MenuItem; 
 
 
public class Analysis extends ActionBarActivity { 
 
    @Override 
    protected void onCreate(Bundle savedInstanceState) { 
        super.onCreate(savedInstanceState); 
        setContentView(R.layout.activity_analysis); 
    } 
 
    @Override 
    public boolean onCreateOptionsMenu(Menu menu) { 
        // Inflate the menu; this adds items to the action bar if it is present. 
        getMenuInflater().inflate(R.menu.menu_analysis, menu); 
        return true; 
    } 
 
    @Override 
    public boolean onOptionsItemSelected(MenuItem item) { 
        // Handle action bar item clicks here. The action bar will 
        // automatically handle clicks on the Home/Up button, so long 
        // as you specify a parent activity in AndroidManifest.xml. 
        int id = item.getItemId(); 
 
        //noinspection SimplifiableIfStatement 
        if (id == R.id.action_settings) { 
            return true; 
        } 
 
        return super.onOptionsItemSelected(item); 
    } 
}
```
