# Supplementary material for: A paper-based, cell-free biosensor system for the detection of heavy metals and date rape drugs
Source: PLoS One. 2019 Mar 6;14(3):e0210940. doi: 10.1371/journal.pone.0210940 (PMC6402643; doi:10.1371/journal.pone.0210940)
Supplement: S2 File — (ZIP) [file pone.0210940.s016.zip › exportToHTMLres/de/anna/cellfreestick/ApplicationTest.java.html]

ApplicationTest.java


|  |
| --- |
| ApplicationTest.java |

```
package de.anna.cellfreestick; 
 
import android.app.Application; 
import android.test.ApplicationTestCase; 
 
/** 
 * <a href="http://d.android.com/tools/testing/testing_android.html">Testing Fundamentals</a> 
 */ 
public class ApplicationTest extends ApplicationTestCase<Application> { 
    public ApplicationTest() { 
        super(Application.class); 
    } 
}
```
