# Supplementary material for: A paper-based, cell-free biosensor system for the detection of heavy metals and date rape drugs
Source: PLoS One. 2019 Mar 6;14(3):e0210940. doi: 10.1371/journal.pone.0210940 (PMC6402643; doi:10.1371/journal.pone.0210940)
Supplement: S2 File — (ZIP) [file pone.0210940.s016.zip › exportToHTMLres/de/anna/cellfreestick/ContaminationList.java.html]

ContaminationList.java


|  |
| --- |
| ContaminationList.java |

```
package de.anna.cellfreestick; 
 
import android.content.DialogInterface; 
import android.content.Intent; 
import android.support.v7.app.ActionBarActivity; 
import android.os.Bundle; 
import android.view.Menu; 
import android.view.MenuItem; 
import android.view.View; 
import android.widget.Button; 
import android.widget.TableLayout; 
import android.widget.TextView; 
 
import java.util.Arrays; 
 
 
public class ContaminationList extends ActionBarActivity implements View.OnClickListener { 
 
    //declaration of variables 
    private int [] medianpos; 
    private int [] medianneg; 
    private boolean [] contamination; 
    private TextView textViewException; 
    private TableLayout contaminationTable; 
    private Button details; 
    private Button about; 
 
    @Override 
    protected void onCreate(Bundle savedInstanceState) { 
        super.onCreate(savedInstanceState); 
        setContentView(R.layout.activity_contamination_list); 
 
        //find elements from View 
        details =(Button) findViewById(R.id.buttonDetails); 
        details.setOnClickListener(this); 
        about=(Button) findViewById(R.id.buttonAbout); 
        about.setOnClickListener(this); 
        textViewException =(TextView) findViewById(R.id.textViewException); 
        contaminationTable = (TableLayout) findViewById(R.id.contaminationTable); 
 
        //store values from intent in variables for further calculations 
        Intent intent = getIntent(); 
        if(intent!=null){ 
            medianpos= intent.getIntArrayExtra("Keypos"); 
            medianneg= intent.getIntArrayExtra("Keyneg"); 
        } 
 
        //call method for the calculation of contamination 
        calculateContamination(); 
 
        //if contamination[false] remove corresponding heavy metal from table view 
       for(int i=6; i>=0; i--) { 
            if(!contamination[i]) contaminationTable.removeViewAt(i); 
        } 
    } 
 
    //method for the calculation of contamination 
    private void calculateContamination() { 
        contamination = new boolean[7]; 
        // check if positive control has a reasonable value, if not throw exception 
        if(medianneg[0]>=20){ 
 
            /* calculation of correction factor because of hazardous metals in the sample 
            that could possibly lead to decrease in fluorescence 
             */ 
 
            float corr = medianneg[0]/medianpos[0]; //relative decrease of fluorescence in positive controls 
 
            /*does the test reach more than ten percent of the positive control? 
            if that is the case the value corresponding to the certain heavy metal is set to 'true' in 
            contamination[] otherwise it is set to 'false' 
             */ 
 
            for(int i=0; i<7; i++){ 
 
                float diff = corr*medianpos[i+1]-medianneg[i+1]; 
                float cont = diff/medianneg[0]; 
                if(cont<0.1 || diff<0){ 
                    contamination[i]=false; 
                }else{ 
                    contamination[i]=true; 
                } 
            } 
            textViewException.setText("Your water is contaminated with:"); 
        }else{ 
            textViewException.setText("The test is not working properly, sorry!"); 
            //Supress showing of heavy metals in table view 
            for(int i=7; i<7; i--) { 
             contaminationTable.removeViewAt(i); 
            } 
        } 
 
 
    } 
 
    @Override 
    public boolean onCreateOptionsMenu(Menu menu) { 
        // Inflate the menu; this adds items to the action bar if it is present. 
        getMenuInflater().inflate(R.menu.menu_contamination_list, menu); 
        return true; 
    } 
 
    @Override 
    public boolean onOptionsItemSelected(MenuItem item) { 
        // Handle action bar item clicks here. The action bar will 
        // automatically handle clicks on the Home/Up button, so long 
        // as you specify a parent activity in AndroidManifest.xml. 
        int id = item.getItemId(); 
 
        //noinspection SimplifiableIfStatement 
        if (id == R.id.action_settings) { 
            return true; 
        } 
 
        return super.onOptionsItemSelected(item); 
    } 
 
    @Override 
    public void onClick(View v) { 
        if(v.getId() == R.id.buttonDetails){ 
 
            Intent intent = new Intent(ContaminationList.this, Results.class); 
            startActivity(intent); 
 
        }else if(v.getId() == R.id.buttonAbout){ 
 
            Intent intent = new Intent (ContaminationList.this, Analysis.class); 
            startActivity(intent); 
 
        } 
 
    } 
}
```
