# Supplementary material for: A paper-based, cell-free biosensor system for the detection of heavy metals and date rape drugs
Source: PLoS One. 2019 Mar 6;14(3):e0210940. doi: 10.1371/journal.pone.0210940 (PMC6402643; doi:10.1371/journal.pone.0210940)
Supplement: S2 File — (ZIP) [file pone.0210940.s016.zip › exportToHTMLres/de/anna/cellfreestick/HeavyMetals.java.html]

HeavyMetals.java


|  |
| --- |
| HeavyMetals.java |

```
package de.anna.cellfreestick; 
 
/** 
 * Created by iGEM-Bielefeld on 30.08.2015. 
 */ 
public class HeavyMetals { 
 
    //declaration of variables 
    private String title; 
    private String content; 
 
    //constructs a simple list element for one of our analytes 
    public HeavyMetals(String title, String content) { 
        this.title = title; 
        this.content = content; 
    } 
 
    public String getTitle() { 
        return title; 
    } 
 
    public void setTitle(String title) { 
        this.title = title; 
    } 
 
    public String getContent() { 
        return content; 
    } 
 
    public void setContent(String content) { 
        this.content = content; 
    } 
 
    @Override 
    public String toString() { 
        return title; 
    } 
}
```
