# Supplementary material for: A paper-based, cell-free biosensor system for the detection of heavy metals and date rape drugs
Source: PLoS One. 2019 Mar 6;14(3):e0210940. doi: 10.1371/journal.pone.0210940 (PMC6402643; doi:10.1371/journal.pone.0210940)
Supplement: S2 File — (ZIP) [file pone.0210940.s016.zip › exportToHTMLres/de/anna/cellfreestick/HeavyMetalsDetailsFragment.java.html]

HeavyMetalsDetailsFragment.java


|  |
| --- |
| HeavyMetalsDetailsFragment.java |

```
package de.anna.cellfreestick; 
 
 
import android.os.Bundle; 
import android.app.Fragment; 
import android.view.LayoutInflater; 
import android.view.View; 
import android.view.ViewGroup; 
import android.widget.TextView; 
 
 
/** 
 * A simple {@link Fragment} subclass. 
 * Use the {@link HeavyMetalsDetailsFragment#newInstance} factory method to 
 * create an instance of this fragment. 
 */ 
 
//default Fragment 
public class HeavyMetalsDetailsFragment extends Fragment { 
    // the fragment initialization parameters, e.g. ARG_ITEM_NUMBER 
    private static final String ARG_PARAM1 = "param1"; 
    private static final String ARG_PARAM2 = "param2"; 
 
 
    private String mParam1; 
    private String mParam2; 
 
 
    public static HeavyMetalsDetailsFragment newInstance(String param1, String param2) { 
        HeavyMetalsDetailsFragment fragment = new HeavyMetalsDetailsFragment(); 
        Bundle args = new Bundle(); 
        args.putString(ARG_PARAM1, param1); 
        args.putString(ARG_PARAM2, param2); 
        fragment.setArguments(args); 
        return fragment; 
    } 
 
    public HeavyMetalsDetailsFragment() { 
        // Required empty public constructor 
    } 
 
    @Override 
    public void onCreate(Bundle savedInstanceState) { 
        super.onCreate(savedInstanceState); 
        if (getArguments() != null) { 
            mParam1 = getArguments().getString(ARG_PARAM1); 
            mParam2 = getArguments().getString(ARG_PARAM2); 
        } 
    } 
 
    @Override 
    public View onCreateView(LayoutInflater inflater, ViewGroup container, 
                             Bundle savedInstanceState) { 
        // Inflate the layout for this fragment 
        return inflater.inflate(R.layout.fragment_heavy_metals_details, container, false); 
    } 
 
}
```
