# Supplementary material for: A paper-based, cell-free biosensor system for the detection of heavy metals and date rape drugs
Source: PLoS One. 2019 Mar 6;14(3):e0210940. doi: 10.1371/journal.pone.0210940 (PMC6402643; doi:10.1371/journal.pone.0210940)
Supplement: S2 File — (ZIP) [file pone.0210940.s016.zip › exportToHTMLres/de/anna/cellfreestick/HeavyMetalsFragment.java.html]

HeavyMetalsFragment.java


|  |
| --- |
| HeavyMetalsFragment.java |

```
package de.anna.cellfreestick; 
 
import android.app.Activity; 
import android.content.Intent; 
import android.os.Bundle; 
import android.app.ListFragment; 
import android.view.LayoutInflater; 
import android.view.View; 
import android.view.ViewGroup; 
import android.widget.ArrayAdapter; 
import android.widget.ListView; 
 
 
import java.util.ArrayList; 
import java.util.List; 
 
 
/** 
 * A fragment representing a list of Items. 
 * Activities containing this fragment MUST implement the {@link OnFragmentInteractionListener} 
 * interface. 
 */ 
 
//default list fragment 
public class HeavyMetalsFragment extends ListFragment { 
 
   // declaration of variables 
    private static final String ARG_PARAM1 = "param1"; 
    private static final String ARG_PARAM2 = "param2"; 
 
    private String mParam1; 
    private String mParam2; 
    private OnFragmentInteractionListener mListener; 
 
 
    //establishment of a new instance of the List 
    public static HeavyMetalsFragment newInstance(String param1, String param2) { 
        HeavyMetalsFragment fragment = new HeavyMetalsFragment(); 
        Bundle args = new Bundle(); 
        args.putString(ARG_PARAM1, param1); 
        args.putString(ARG_PARAM2, param2); 
        fragment.setArguments(args); 
        return fragment; 
    } 
 
    /** 
     * Mandatory empty constructor for the fragment manager to instantiate the 
     * fragment (e.g. upon screen orientation changes). 
     */ 
    public HeavyMetalsFragment() { 
    } 
 
    @Override 
    public void onCreate(Bundle savedInstanceState) { 
        super.onCreate(savedInstanceState); 
 
        if (getArguments() != null) { 
            mParam1 = getArguments().getString(ARG_PARAM1); 
            mParam2 = getArguments().getString(ARG_PARAM2); 
        } 
 
        //creation of a new list with HeavyMetal instances 
        List<HeavyMetals> heavyMetalsList = new ArrayList<HeavyMetals>(); 
 
        //hardcoded filling of the list with our analytes 
        heavyMetalsList.add(new HeavyMetals("Date rape drugs","a")); 
        heavyMetalsList.add(new HeavyMetals("Arsenic", "b")); 
        heavyMetalsList.add(new HeavyMetals("Mercury","c")); 
        heavyMetalsList.add(new HeavyMetals("Chromium","d")); 
        heavyMetalsList.add(new HeavyMetals("Lead","e")); 
        heavyMetalsList.add(new HeavyMetals("Nickel","f")); 
        heavyMetalsList.add(new HeavyMetals("Copper","g")); 
 
        //set list adapter 
       setListAdapter(new ArrayAdapter<HeavyMetals>(getActivity(), 
        R.layout.simple_list_item, android.R.id.text1, heavyMetalsList)); 
    } 
 
 
    @Override 
    public void onAttach(Activity activity) { 
        super.onAttach(activity); 
        try { 
            mListener = (OnFragmentInteractionListener) activity; 
        } catch (ClassCastException e) { 
            throw new ClassCastException(activity.toString() 
                    + " must implement OnFragmentInteractionListener"); 
        } 
    } 
 
    @Override 
    public void onDetach() { 
        super.onDetach(); 
        mListener = null; 
    } 
 
    @Override 
    public void onListItemClick(ListView l, View v, int position, long id) { 
        super.onListItemClick(l, v, position, id); 
 
        //addition of title to intent 
        Intent intent = new Intent(getActivity(),HeavyMetalsDetailsActivity.class); 
        String title = l.getItemAtPosition(position).toString(); 
        intent.putExtra("value_title", title); 
        getActivity().startActivity(intent); 
 
        if (null != mListener) { 
            // Notify the active callbacks interface (the activity, if the 
            // fragment is attached to one) that an item has been selected. 
            //mListener.onFragmentInteraction(DummyContent.ITEMS.get(position).id); 
        } 
    } 
 
    /** 
     * This interface must be implemented by activities that contain this 
     * fragment to allow an interaction in this fragment to be communicated 
     * to the activity and potentially other fragments contained in that 
     * activity. 
     * <p/> 
     * See the Android Training lesson <a href= 
     * "http://developer.android.com/training/basics/fragments/communicating.html" 
     * >Communicating with Other Fragments</a> for more information. 
     */ 
    public interface OnFragmentInteractionListener { 
 
        public void onFragmentInteraction(String id); 
    } 
 
    @Override 
    public View onCreateView(LayoutInflater inflater, ViewGroup container, Bundle savedInstanceState) { 
        return inflater.inflate(R.layout.fragment_heavy_metals, container, false); 
    } 
}
```
