# Supplementary material for: A paper-based, cell-free biosensor system for the detection of heavy metals and date rape drugs
Source: PLoS One. 2019 Mar 6;14(3):e0210940. doi: 10.1371/journal.pone.0210940 (PMC6402643; doi:10.1371/journal.pone.0210940)
Supplement: S2 File — (ZIP) [file pone.0210940.s016.zip › exportToHTMLres/de/anna/cellfreestick/MainActivity.java.html]

MainActivity.java


|  |
| --- |
| MainActivity.java |

```
package de.anna.cellfreestick; 
 
import android.app.ActionBar; 
import android.app.Activity; 
import android.content.ContentValues; 
import android.content.Intent; 
import android.graphics.Bitmap; 
import android.net.Uri; 
import android.os.Environment; 
import android.provider.MediaStore; 
import android.support.v7.app.ActionBarActivity; 
import android.os.Bundle; 
import android.util.Log; 
import android.view.Menu; 
import android.view.MenuItem; 
import android.view.View; 
import android.view.View.OnClickListener; 
import android.widget.Button; 
import android.widget.ImageView; 
 
import java.io.File; 
import java.io.IOException; 
 
 
public class MainActivity extends Activity implements OnClickListener { 
 
    public Button buttonInstructions; 
    public Button buttonTakePhoto; 
 
 
 
    @Override 
    public void onCreate(Bundle savedInstanceState) { 
        super.onCreate(savedInstanceState); 
        setContentView(R.layout.activity_main); 
 
        //find View elements 
        buttonInstructions = (Button)findViewById(R.id.buttonInstructions); 
        buttonTakePhoto = (Button)findViewById(R.id.buttonTakePhoto); 
        buttonInstructions.setOnClickListener (this); 
        buttonTakePhoto.setOnClickListener(this); 
 
 
    } 
 
    //define intents for buttons 
    public void onClick(View v){ 
        if(v.getId() == R.id.buttonInstructions){ 
            Intent intent = new Intent(MainActivity.this, Instructions.class); 
            startActivity(intent); 
 
        }else if(v.getId() == R.id.buttonTakePhoto){ 
 
            Intent intent = new Intent (MainActivity.this, TakePhoto.class); 
            startActivity(intent); 
 
        } 
    } 
 
    @Override 
    public boolean onCreateOptionsMenu(Menu menu) { 
        // Inflate the menu; this adds items to the action bar if it is present. 
        getMenuInflater().inflate(R.menu.menu_main, menu); 
        return true; 
    } 
 
 
 
 
         
 
    @Override 
    public boolean onOptionsItemSelected(MenuItem item) { 
        // Handle action bar item clicks here. The action bar will 
        // automatically handle clicks on the Home/Up button, so long 
        // as you specify a parent activity in AndroidManifest.xml. 
        int id = item.getItemId(); 
 
        //noinspection SimplifiableIfStatement 
        if (id == R.id.action_settings) { 
            return true; 
        } 
 
        return super.onOptionsItemSelected(item); 
    } 
 
 
 
}
```
