# Supplementary material for: A paper-based, cell-free biosensor system for the detection of heavy metals and date rape drugs
Source: PLoS One. 2019 Mar 6;14(3):e0210940. doi: 10.1371/journal.pone.0210940 (PMC6402643; doi:10.1371/journal.pone.0210940)
Supplement: S2 File — (ZIP) [file pone.0210940.s016.zip › exportToHTMLres/de/anna/cellfreestick/TakePhoto.java.html]

TakePhoto.java


|  |
| --- |
| TakePhoto.java |

```
package de.anna.cellfreestick; 
 
import android.content.ContentValues; 
import android.content.Intent; 
import android.graphics.Bitmap; 
import android.graphics.Color; 
import android.graphics.PixelFormat; 
import android.net.Uri; 
import android.provider.MediaStore; 
import android.support.v7.app.ActionBarActivity; 
import android.os.Bundle; 
import android.util.Log; 
import android.view.Menu; 
import android.view.MenuItem; 
import android.view.View; 
import android.widget.Button; 
import android.widget.ImageView; 
import android.widget.ListView; 
import android.widget.TextView; 
 
import java.io.IOException; 
import java.lang.reflect.Array; 
import java.util.Arrays; 
 
import static android.graphics.Color.parseColor; 
 
 
public class TakePhoto extends ActionBarActivity implements View.OnClickListener{ 
 
    //declaration of variables 
    private Button buttonResults; 
    private ImageView imageViewPhoto; 
    private TextView pixelanzeige; 
    private Uri imageUri; 
    private static final String TITLE = "TakePhoto"; 
    private static final String DESCRIPTION = "Foto taken by App"; 
    private static final String TAG = MainActivity.class.getSimpleName(); 
    private static final int IMAGE_CAPTURE = 1; 
    private Bitmap bitmap3; 
    private int [] medianpos = new int[8]; 
    private int [] medianneg = new int[8]; 
 
    @Override 
    protected void onCreate(Bundle savedInstanceState) { 
        super.onCreate(savedInstanceState); 
        setContentView(R.layout.activity_take_photo); 
 
        //find view elements 
        buttonResults = (Button) findViewById(R.id.buttonResults); 
        buttonResults.setOnClickListener(this); 
        imageViewPhoto = (ImageView) findViewById(R.id.imageViewPhoto); 
 
        //take a picture and save it 
        startCamera(); 
 
    } 
 
    //starts the camera 
    private void startCamera(){ 
        ContentValues values = new ContentValues(); 
        values.put(MediaStore.Images.Media.TITLE, TITLE); 
        values.put(MediaStore.Images.Media.DESCRIPTION, DESCRIPTION); 
        values.put(MediaStore.Images.Media.MIME_TYPE, "image/jpeg"); 
        imageUri = getContentResolver().insert( 
                MediaStore.Images.Media.EXTERNAL_CONTENT_URI, values); 
        Intent intent = new Intent(MediaStore.ACTION_IMAGE_CAPTURE); 
        intent.putExtra(MediaStore.EXTRA_OUTPUT, imageUri); 
        startActivityForResult(intent, IMAGE_CAPTURE); 
    } 
 
    @Override 
    protected void onActivityResult(int requestCode, int resultCode, Intent data) { 
        super.onActivityResult(requestCode, resultCode, data); 
        if (requestCode == IMAGE_CAPTURE) { 
            if (resultCode == RESULT_OK) { 
                try { 
 
                    //create Bitmap from saved picture 
                    Bitmap bitmapRRR = MediaStore.Images.Media.getBitmap( 
                            getContentResolver(), imageUri); 
                    float w1 = bitmapRRR.getWidth(); 
                    float h1 = bitmapRRR.getHeight(); 
                    int h2 = 300; 
                    int w2 = (int) (w1 / h1 * (float) h2); 
                    bitmap3 = Bitmap.createScaledBitmap(bitmapRRR, w2, h2, false); 
 
                    //calculate the green values of examined pixels 
                    calculationGreen(); 
 
                    //show altered picture 
                    imageViewPhoto.setImageBitmap(bitmap3); 
 
                } catch (IOException e) { 
                    Log.e(TAG, "setBitmap()", e); 
                } 
            } else { 
                int rowsDeleted = getContentResolver().delete(imageUri, null, null); 
                Log.d(TAG, rowsDeleted + " rows deleted"); 
            } 
        } 
 
    } 
 
    //calculation of the median of the green values of the examined pixels 
    private void calculationGreen() { 
        int[] pixelwerte = new int[100]; 
        int[] pixelwerteGreen = new int[100]; 
        int l1=0; 
        int l2=0; 
 
 
        for (int n = 1; n < 4; n = n + 2){ 
            for (int m = 1; m < 9; m++) { 
                int k = 0; 
                for (int i = 0; i < 10; i++) { 
                    for (int j = 0; j < 10; j++) { 
                        pixelwerte[k] = bitmap3.getPixel(m * (bitmap3.getWidth() / 9) + i, n * (bitmap3.getHeight() / 4) + j); 
                        pixelwerteGreen[k] = Color.green(pixelwerte[k]); 
                        k++; 
                        bitmap3.setPixel(m * (bitmap3.getWidth() / 9) + i, n * (bitmap3.getHeight() / 4) + j, 0xffff0000); 
                    } 
                } 
 
                //calculation of median of green values 
                Arrays.sort(pixelwerteGreen); 
                if (n == 1){ medianpos[l1] = pixelwerteGreen[pixelwerteGreen.length / 2]; 
                    l1++; 
                }else {medianneg[l2]=pixelwerteGreen[pixelwerteGreen.length/2];l2++;} 
 
            } 
    } 
 
        pixelanzeige = (TextView) findViewById(R.id.pixelanzeige); 
        pixelanzeige.setText("Please check if the red square in the left bottom corner is inside the green circle! If this is not the case, take a new picture. The calculated values for the green color of the spots: " + Arrays.toString(medianpos) + Arrays.toString(medianneg)); 
 
    } 
 
    @Override 
    public boolean onCreateOptionsMenu(Menu menu) { 
        // Inflate the menu; this adds items to the action bar if it is present. 
        getMenuInflater().inflate(R.menu.menu_take_photo, menu); 
        return true; 
    } 
 
    @Override 
    public boolean onOptionsItemSelected(MenuItem item) { 
        // Handle action bar item clicks here. The action bar will 
        // automatically handle clicks on the Home/Up button, so long 
        // as you specify a parent activity in AndroidManifest.xml. 
        int id = item.getItemId(); 
 
        //noinspection SimplifiableIfStatement 
        if (id == R.id.action_settings) { 
            return true; 
        } 
 
        return super.onOptionsItemSelected(item); 
    } 
 
    @Override 
    public void onClick(View view) { 
        Intent intent = new Intent(this, ContaminationList.class); 
        //String inhalt = Arrays.toString(medianpos) + Arrays.toString(medianneg); 
        intent.putExtra("Keyneg", medianneg); 
        intent.putExtra("Keypos", medianpos); 
 
        startActivity(intent); 
    } 
 
 
}
```
