# Supplementary material for: A paper-based, cell-free biosensor system for the detection of heavy metals and date rape drugs
Source: PLoS One. 2019 Mar 6;14(3):e0210940. doi: 10.1371/journal.pone.0210940 (PMC6402643; doi:10.1371/journal.pone.0210940)
Supplement: S2 File — (ZIP) [file pone.0210940.s016.zip › exportToHTMLres/layout/activity_analysis.xml.html]

activity\_analysis.xml


|  |
| --- |
| activity\_analysis.xml |

```
<RelativeLayout xmlns:android="http://schemas.android.com/apk/res/android" 
    xmlns:tools="http://schemas.android.com/tools" android:layout_width="match_parent" 
    android:layout_height="match_parent" android:paddingLeft="@dimen/activity_horizontal_margin" 
    android:paddingRight="@dimen/activity_horizontal_margin" 
    android:paddingTop="@dimen/activity_vertical_margin" 
    android:paddingBottom="@dimen/activity_vertical_margin" 
    tools:context="de.anna.cellfreestick.Analysis" 
    android:background="#ff322f32" 
    style="@style/Base.Theme.AppCompat"> 
 
    <TextView android:text="@string/analysis" android:layout_width="fill_parent" 
        android:layout_height="fill_parent" 
        android:id="@+id/textAnalysis" 
        android:gravity="center_horizontal" 
        android:textColor="#ffffffff"/> 
 
    <ScrollView 
        android:layout_width="wrap_content" 
        android:layout_height="wrap_content" 
        android:id="@+id/scrollView" 
        android:layout_alignParentBottom="true" /> 
 
</RelativeLayout>
```
