# Supplementary material for: A paper-based, cell-free biosensor system for the detection of heavy metals and date rape drugs
Source: PLoS One. 2019 Mar 6;14(3):e0210940. doi: 10.1371/journal.pone.0210940 (PMC6402643; doi:10.1371/journal.pone.0210940)
Supplement: S2 File — (ZIP) [file pone.0210940.s016.zip › exportToHTMLres/layout/activity_contamination_list.xml.html]

activity\_contamination\_list.xml


|  |
| --- |
| activity\_contamination\_list.xml |

```
<RelativeLayout xmlns:android="http://schemas.android.com/apk/res/android" 
    xmlns:tools="http://schemas.android.com/tools" android:layout_width="match_parent" 
    android:layout_height="match_parent" android:paddingLeft="@dimen/activity_horizontal_margin" 
    android:paddingRight="@dimen/activity_horizontal_margin" 
    android:paddingTop="@dimen/activity_vertical_margin" 
    android:paddingBottom="@dimen/activity_vertical_margin" 
    tools:context="de.anna.cellfreestick.ContaminationList" 
    style="@style/Base.Theme.AppCompat" 
    android:background="#ff322f32" 
     > 
 
    <TextView 
        android:textColor="#ffffffff" 
        android:layout_width="wrap_content" 
        android:layout_height="wrap_content" 
        android:textAppearance="?android:attr/textAppearanceLarge" 
        android:text="@string/title_activity_contamination_list" 
        android:id="@+id/textViewContaminationList" 
        android:layout_column="1" 
        android:layout_alignParentTop="true" 
        android:layout_centerHorizontal="true" 
        android:textSize="60sp" 
        android:textStyle="bold" /> 
 
    <TableLayout 
        android:layout_width="match_parent" 
        android:layout_height="match_parent" 
        android:layout_alignParentStart="true" 
        android:id="@+id/contaminationTable" 
        android:layout_alignParentBottom="true" 
        android:layout_below="@+id/textViewException"> 
 
 
        <TableRow 
            android:layout_width="match_parent" 
            android:layout_height="match_parent" 
            android:id="@+id/dateRapeDrugs"> 
 
            <TextView 
                android:textColor="#ffffffff" 
                android:layout_width="wrap_content" 
                android:layout_height="wrap_content" 
                android:text= "Date rape drugs" 
                android:textSize="30sp" 
                android:id="@+id/textViewDateRapeDrugs" 
                android:layout_column="2" 
                /> 
 
            <ImageView 
                android:layout_width="wrap_content" 
                android:layout_height="wrap_content" 
                android:id="@+id/imageViewDateRapeDrugs" 
                android:layout_column="1"/> 
        </TableRow> 
 
        <TableRow 
            android:layout_width="match_parent" 
            android:layout_height="match_parent" 
            android:id="@+id/arsenic"> 
 
            <TextView 
                android:textColor="#ffffffff" 
                android:layout_width="wrap_content" 
                android:layout_height="wrap_content" 
                android:text="Arsenic" 
                android:textSize="30sp" 
                android:layout_column="2" 
                android:id="@+id/textViewArsenic" /> 
 
            <ImageView 
                android:layout_width="wrap_content" 
                android:layout_height="wrap_content" 
                android:layout_column="1" 
                android:id="@+id/imageViewArsenic" /> 
        </TableRow> 
 
        <TableRow 
            android:layout_width="match_parent" 
            android:layout_height="match_parent"> 
 
            <TextView 
                android:textColor="#ffffffff" 
                android:layout_width="wrap_content" 
                android:layout_height="wrap_content" 
                android:text="Mercury" 
                android:textSize="30sp" 
                android:layout_column="2" 
                android:id="@+id/textViewMercury" /> 
 
            <ImageView 
                android:layout_width="wrap_content" 
                android:layout_height="wrap_content" 
                android:layout_column="1" 
                android:id="@+id/imageViewMercury" /> 
        </TableRow> 
 
        <TableRow 
            android:layout_width="match_parent" 
            android:layout_height="match_parent"> 
 
            <TextView 
                android:textColor="#ffffffff" 
                android:layout_width="wrap_content" 
                android:layout_height="wrap_content" 
                android:text="Chromium" 
                android:textSize="30sp" 
                android:layout_column="2" 
                android:id="@+id/textViewChromium" /> 
 
            <ImageView 
                android:layout_width="wrap_content" 
                android:layout_height="wrap_content" 
                android:layout_column="1" 
                android:id="@+id/imageViewChromium" /> 
        </TableRow> 
 
        <TableRow 
            android:layout_width="match_parent" 
            android:layout_height="match_parent"> 
 
            <TextView 
                android:textColor="#ffffffff" 
                android:layout_width="wrap_content" 
                android:layout_height="wrap_content" 
                android:text="Lead" 
                android:textSize="30sp" 
                android:layout_column="2" 
                android:id="@+id/textViewLead" /> 
 
            <ImageView 
                android:layout_width="wrap_content" 
                android:layout_height="wrap_content" 
                android:layout_column="1" 
                android:id="@+id/imageViewLead" /> 
        </TableRow> 
 
        <TableRow 
            android:layout_width="match_parent" 
            android:layout_height="match_parent"> 
 
            <TextView 
                android:textColor="#ffffffff" 
                android:layout_width="wrap_content" 
                android:layout_height="wrap_content" 
                android:text="Nickel" 
                android:textSize="30sp" 
                android:layout_column="2" 
                android:id="@+id/textViewNickel" /> 
 
            <ImageView 
                android:layout_width="wrap_content" 
                android:layout_height="wrap_content" 
                android:layout_column="1" 
                android:id="@+id/imageViewNickel" /> 
        </TableRow> 
 
        <TableRow 
            android:layout_width="match_parent" 
            android:layout_height="match_parent"> 
 
            <TextView 
                android:textColor="#ffffffff" 
                android:layout_width="wrap_content" 
                android:layout_height="wrap_content" 
                android:text="Copper" 
                android:textSize="30sp" 
                android:layout_column="2" 
                android:id="@+id/textViewCopper" /> 
 
            <ImageView 
                android:layout_width="wrap_content" 
                android:layout_height="wrap_content" 
                android:layout_column="1" 
                android:id="@+id/imageViewCopper" /> 
        </TableRow> 
    </TableLayout> 
 
    <TextView 
        android:textColor="#ffffffff" 
        android:textSize="30sp" 
        android:layout_width="wrap_content" 
        android:layout_height="wrap_content" 
        android:text="May you always drink the purest of all waters!" 
        android:id="@+id/textViewException" 
        android:layout_alignParentEnd="true" 
        android:layout_below="@+id/textViewContaminationList" /> 
 
    <Button 
        android:layout_width="wrap_content" 
        android:layout_height="wrap_content" 
        android:text="About" 
        android:id="@+id/buttonAbout" 
        android:layout_alignParentBottom="true" 
        android:layout_alignParentLeft="true" 
        android:textColor="#ffffffff" 
        android:textStyle="bold" 
        android:background="#ffe31918" 
        android:minWidth="140dp" 
        android:clickable="true" /> 
 
    <Button 
        android:layout_width="wrap_content" 
        android:layout_height="wrap_content" 
        android:text="Details" 
        android:id="@+id/buttonDetails" 
        android:layout_alignParentBottom="true" 
        android:layout_alignParentRight="true" 
        android:textColor="#ffffffff" 
        android:textStyle="bold" 
        android:background="#ffe31918" 
        android:minWidth="140dp" 
        android:clickable="true" /> 
 
</RelativeLayout>
```
