# Supplementary material for: A paper-based, cell-free biosensor system for the detection of heavy metals and date rape drugs
Source: PLoS One. 2019 Mar 6;14(3):e0210940. doi: 10.1371/journal.pone.0210940 (PMC6402643; doi:10.1371/journal.pone.0210940)
Supplement: S2 File — (ZIP) [file pone.0210940.s016.zip › exportToHTMLres/layout/activity_heavy_metals_details.xml.html]

activity\_heavy\_metals\_details.xml


|  |
| --- |
| activity\_heavy\_metals\_details.xml |

```
<RelativeLayout xmlns:android="http://schemas.android.com/apk/res/android" 
    xmlns:tools="http://schemas.android.com/tools" android:layout_width="match_parent" 
    android:layout_height="match_parent" 
    tools:context="de.anna.cellfreestick.HeavyMetalsDetailsActivity" 
    android:background="#ff322f32" 
    style="@style/Base.Theme.AppCompat"> 
 
    <fragment 
        android:layout_width="match_parent" 
        android:layout_height="match_parent" 
        android:name="de.anna.cellfreestick.HeavyMetalsDetailsFragment" 
        android:id="@+id/fragment" 
        android:layout_centerVertical="true" 
        android:layout_centerHorizontal="true" 
        tools:layout="@layout/fragment_heavy_metals_details" /> 
</RelativeLayout>
```
