# Supplementary material for: A paper-based, cell-free biosensor system for the detection of heavy metals and date rape drugs
Source: PLoS One. 2019 Mar 6;14(3):e0210940. doi: 10.1371/journal.pone.0210940 (PMC6402643; doi:10.1371/journal.pone.0210940)
Supplement: S2 File — (ZIP) [file pone.0210940.s016.zip › exportToHTMLres/layout/activity_instructions.xml.html]

activity\_instructions.xml


|  |
| --- |
| activity\_instructions.xml |

```
<RelativeLayout xmlns:android="http://schemas.android.com/apk/res/android" 
    xmlns:tools="http://schemas.android.com/tools" android:layout_width="match_parent" 
    android:layout_height="match_parent" tools:context="de.anna.cellfreestick.Instructions" 
    android:background="#ff322f32" 
    style="@style/Base.Theme.AppCompat"> 
 
 
 
    <TextView 
        android:layout_width="wrap_content" 
        android:layout_height="wrap_content" 
        android:textAppearance="?android:attr/textAppearanceLarge" 
        android:text="Instructions" 
        android:id="@+id/textViewInstruction" 
        android:layout_alignParentTop="true" 
        android:layout_centerHorizontal="true" 
        android:textSize="60sp" 
        android:textColor="#ffffffff" 
        android:textStyle="bold" /> 
 
    <TextView 
        android:layout_width="wrap_content" 
        android:layout_height="wrap_content" 
        android:textAppearance="?android:attr/textAppearanceLarge" 
        android:text="@string/textInstruction1" 
        android:id="@+id/textInstruction1" 
        android:layout_alignParentTop="true" 
        android:layout_centerHorizontal="true" 
        android:textSize="30sp" 
        android:layout_marginTop="80dp" 
        android:layout_marginLeft="20dp" 
        android:layout_marginRight="20dp" 
        android:textColor="#ffffffff" 
        android:textIsSelectable="false" 
        android:textStyle="bold" /> 
 
    <TextView 
        android:layout_width="wrap_content" 
        android:layout_height="wrap_content" 
        android:textAppearance="?android:attr/textAppearanceLarge" 
        android:text="@string/textInstruction2" 
        android:id="@+id/textInstruction2" 
        android:layout_below="@+id/textInstruction1" 
        android:layout_centerHorizontal="true" 
        android:textSize="30sp" 
        android:layout_marginTop="15dp" 
        android:layout_marginLeft="20dp" 
        android:layout_marginRight="20dp" 
        android:textColor="#ffffffff" 
        android:textStyle="bold" /> 
 
    <TextView 
        android:layout_width="wrap_content" 
        android:layout_height="wrap_content" 
        android:textAppearance="?android:attr/textAppearanceLarge" 
        android:text="@string/textInstruction3" 
        android:id="@+id/textInstruction3" 
        android:textSize="30sp" 
        android:layout_marginLeft="20dp" 
        android:layout_marginRight="20dp" 
        android:layout_below="@+id/textInstruction2" 
        android:layout_alignParentStart="true" 
        android:layout_marginTop="20dp" 
        android:textColor="#ffffffff" 
        android:textStyle="bold" /> 
 
    <TextView 
        android:layout_width="wrap_content" 
        android:layout_height="wrap_content" 
        android:textAppearance="?android:attr/textAppearanceLarge" 
        android:text="@string/textInstruction4" 
        android:id="@+id/textInstruction4" 
        android:layout_below="@+id/textInstruction3" 
        android:layout_centerHorizontal="true" 
        android:textSize="30sp" 
        android:layout_marginTop="20dp" 
        android:layout_marginLeft="20dp" 
        android:layout_marginRight="20dp" 
        android:textColor="#ffffffff" 
        android:textStyle="bold" /> 
 
 
 
    <Button 
        android:layout_width="fill_parent" 
        android:layout_height="wrap_content" 
        android:id="@+id/buttonTakePhotoInstructions" 
        android:layout_alignParentBottom="true" 
        android:layout_centerHorizontal="true" 
        android:layout_marginBottom="15dp" 
        android:layout_marginLeft="15dp" 
        android:layout_marginRight="15dp" 
 
        android:text="@string/buttonTakePhotoInstruction" 
        android:height="20sp" 
        android:minWidth="140dp" 
        android:background="#ffe31918" 
        android:textColor="#ffffffff" 
        android:textSize="30sp" 
        android:textStyle="bold" 
        android:clickable="true" /> 
 
</RelativeLayout>
```
