# Supplementary material for: A paper-based, cell-free biosensor system for the detection of heavy metals and date rape drugs
Source: PLoS One. 2019 Mar 6;14(3):e0210940. doi: 10.1371/journal.pone.0210940 (PMC6402643; doi:10.1371/journal.pone.0210940)
Supplement: S2 File — (ZIP) [file pone.0210940.s016.zip › exportToHTMLres/layout/activity_main.xml.html]

activity\_main.xml


|  |
| --- |
| activity\_main.xml |

```
<RelativeLayout xmlns:android="http://schemas.android.com/apk/res/android" 
    xmlns:tools="http://schemas.android.com/tools" android:layout_width="match_parent" 
    android:layout_height="match_parent" android:paddingLeft="@dimen/activity_horizontal_margin" 
    android:paddingRight="@dimen/activity_horizontal_margin" 
    android:paddingTop="@dimen/activity_vertical_margin" 
    android:paddingBottom="@dimen/activity_vertical_margin" tools:context=".MainActivity" 
    android:background="#ff322f32" 
    style="@style/Base.Theme.AppCompat"> 
 
    <ImageView 
        android:layout_width="wrap_content" 
        android:layout_height="wrap_content" 
        android:id="@+id/imageLogo" 
        android:layout_alignParentTop="true" 
        android:layout_centerHorizontal="true" 
        android:src="@mipmap/colilogo" 
        android:contentDescription="@string/imageLogo" /> 
 
    <Button 
        android:layout_width="wrap_content" 
        android:layout_height="wrap_content" 
        android:text="@string/buttonInstruction" 
        android:id="@+id/buttonInstructions" 
        android:layout_alignParentBottom="true" 
        android:layout_alignParentStart="true" 
        android:height="20sp" 
        android:layout_marginLeft="15dp" 
        android:layout_marginBottom="15dp" 
        android:background="#ffe31918" 
        style="@style/Base.ThemeOverlay.AppCompat.Dark" 
        android:ellipsize="end" 
        android:enabled="true" 
        android:elegantTextHeight="true" 
        android:minWidth="140sp" 
        android:textColor="#ffffffff" 
        android:textSize="15sp" 
        android:textStyle="bold" 
        android:clickable="true" /> 
 
    <Button 
        android:layout_width="wrap_content" 
        android:layout_height="wrap_content" 
        android:text="@string/buttonTakePhoto" 
        android:id="@+id/buttonTakePhoto" 
        android:layout_alignParentBottom="true" 
        android:layout_alignParentEnd="true" 
        android:height="10sp" 
        android:layout_marginBottom="15dp" 
        android:layout_marginRight="15dp" 
        android:background="#ffe31918" 
        android:layout_alignParentLeft="false" 
        android:minWidth="140sp" 
        android:textColor="#ffffffff" 
        android:textSize="15sp" 
        android:textStyle="bold" 
        android:clickable="true" /> 
 
</RelativeLayout>
```
