# Supplementary material for: A paper-based, cell-free biosensor system for the detection of heavy metals and date rape drugs
Source: PLoS One. 2019 Mar 6;14(3):e0210940. doi: 10.1371/journal.pone.0210940 (PMC6402643; doi:10.1371/journal.pone.0210940)
Supplement: S2 File — (ZIP) [file pone.0210940.s016.zip › exportToHTMLres/layout/activity_results.xml.html]

activity\_results.xml


|  |
| --- |
| activity\_results.xml |

```
<RelativeLayout xmlns:android="http://schemas.android.com/apk/res/android" 
    xmlns:tools="http://schemas.android.com/tools" android:layout_width="match_parent" 
    android:layout_height="match_parent" android:paddingLeft="@dimen/activity_horizontal_margin" 
    android:paddingRight="@dimen/activity_horizontal_margin" 
    android:paddingTop="@dimen/activity_vertical_margin" 
    android:paddingBottom="@dimen/activity_vertical_margin" 
    tools:context="de.anna.cellfreestick.Results" 
    android:background="#ff322f32" 
    style="@style/Base.Theme.AppCompat"> 
 
 
    <TextView 
        android:layout_width="wrap_content" 
        android:layout_height="wrap_content" 
        android:textAppearance="?android:attr/textAppearanceLarge" 
        android:text="@string/textViewResults" 
        android:id="@+id/textViewResults" 
        android:layout_alignParentTop="true" 
        android:layout_centerHorizontal="true" 
        android:textSize="60sp" 
        android:textColor="#ffffffff" 
        android:textStyle="bold" /> 
 
    <fragment 
        android:layout_width="wrap_content" 
        android:layout_height="wrap_content" 
        android:name="de.anna.cellfreestick.HeavyMetalsFragment" 
        android:id="@+id/fragment2" 
        tools:layout="@android:layout/simple_list_item_1" 
        android:layout_centerInParent="true" 
        android:textColor="#ffffffff" 
         /> 
 
    <ImageView 
        android:id="@+id/icon" 
        android:layout_width="22px" 
        android:layout_height="22px" 
        android:layout_marginLeft="4px" 
        android:layout_marginRight="10px" 
        android:layout_marginTop="4px" 
        android:src="@drawable/logo" > 
    </ImageView> 
 
</RelativeLayout>
```
