# Supplementary material for: A paper-based, cell-free biosensor system for the detection of heavy metals and date rape drugs
Source: PLoS One. 2019 Mar 6;14(3):e0210940. doi: 10.1371/journal.pone.0210940 (PMC6402643; doi:10.1371/journal.pone.0210940)
Supplement: S2 File — (ZIP) [file pone.0210940.s016.zip › exportToHTMLres/layout/activity_take_photo.xml.html]

activity\_take\_photo.xml


|  |
| --- |
| activity\_take\_photo.xml |

```
<RelativeLayout xmlns:android="http://schemas.android.com/apk/res/android" 
    xmlns:tools="http://schemas.android.com/tools" android:layout_width="match_parent" 
    android:layout_height="match_parent" android:paddingLeft="@dimen/activity_horizontal_margin" 
    android:paddingRight="@dimen/activity_horizontal_margin" 
    android:paddingTop="@dimen/activity_vertical_margin" 
    android:paddingBottom="@dimen/activity_vertical_margin" 
    tools:context="de.anna.cellfreestick.TakePhoto" 
    android:background="#ff322f32" 
    style="@style/Base.Theme.AppCompat"> 
 
    <ImageView 
        android:layout_width="match_parent" 
        android:layout_height="match_parent" 
        android:id="@+id/imageViewPhoto" 
        android:layout_alignParentTop="true" 
        android:layout_centerHorizontal="true" 
        android:layout_marginTop="20dp" 
 
        android:layout_marginBottom="300dp" 
        android:focusable="false" 
        android:layout_marginRight="20dp" 
        android:layout_marginLeft="20dp" /> 
 
    <Button 
        android:layout_width="wrap_content" 
        android:layout_height="wrap_content" 
        android:text="@string/buttonResults" 
        android:id="@+id/buttonResults" 
        android:layout_alignParentBottom="true" 
        android:layout_centerHorizontal="true" 
        android:layout_marginBottom="15dp" 
        android:background="#ffe31918" 
        android:height="20sp" 
        android:minWidth="140dp" 
        android:textColor="#ffffffff" 
        android:textStyle="bold" 
        android:clickable="true" /> 
 
    <TextView 
        android:textSize="20sp" 
        android:layout_width="wrap_content" 
        android:layout_height="wrap_content" 
        android:text="New Text" 
        android:id="@+id/pixelanzeige" 
        android:layout_above="@+id/buttonResults" 
        android:textColor="#ffffffff" /> 
</RelativeLayout>
```
