# Supplementary material for: A paper-based, cell-free biosensor system for the detection of heavy metals and date rape drugs
Source: PLoS One. 2019 Mar 6;14(3):e0210940. doi: 10.1371/journal.pone.0210940 (PMC6402643; doi:10.1371/journal.pone.0210940)
Supplement: S2 File — (ZIP) [file pone.0210940.s016.zip › exportToHTMLres/layout/fragment_heavy_metals.xml.html]

fragment\_heavy\_metals.xml


|  |
| --- |
| fragment\_heavy\_metals.xml |

```
<?xml version="1.0" encoding="utf-8"?> 
<LinearLayout xmlns:android="http://schemas.android.com/apk/res/android" 
    android:orientation="vertical" android:layout_width="match_parent" 
    android:layout_height="match_parent" 
    android:background="#ff322f32"> 
 
    <TextView 
        android:layout_width="wrap_content" 
        android:layout_height="wrap_content" 
        android:textAppearance="?android:attr/textAppearanceLarge" 
        android:textSize="40sp" 
        android:textStyle="bold" 
        android:text="Details about:" 
        android:textColor="#ffffffff" 
        android:id="@+id/textViewHeavyMetalsInfo" 
        android:layout_gravity="center_horizontal" /> 
 
    <ListView 
        android:layout_width="match_parent" 
        android:layout_height="match_parent" 
        android:id="@android:id/list" 
        android:divider="#ffffffff" 
        android:dividerHeight="2px" 
        android:layout_marginTop="10dp" 
        android:layout_marginLeft="10dp" 
        android:layout_marginRight="10dp" 
        android:background="#ff322f32" 
        style="@style/Base.Theme.AppCompat"/> 
 
 
 
</LinearLayout>
```
