# Supplementary material for: A paper-based, cell-free biosensor system for the detection of heavy metals and date rape drugs
Source: PLoS One. 2019 Mar 6;14(3):e0210940. doi: 10.1371/journal.pone.0210940 (PMC6402643; doi:10.1371/journal.pone.0210940)
Supplement: S2 File — (ZIP) [file pone.0210940.s016.zip › exportToHTMLres/layout/fragment_heavy_metals_details.xml.html]

fragment\_heavy\_metals\_details.xml


|  |
| --- |
| fragment\_heavy\_metals\_details.xml |

```
<RelativeLayout xmlns:android="http://schemas.android.com/apk/res/android" 
    xmlns:tools="http://schemas.android.com/tools" android:layout_width="match_parent" 
    android:layout_height="match_parent" 
    tools:context="de.anna.cellfreestick.MainActivity" 
    android:gravity="top" 
    android:background="#ff322f32" 
    style="@style/Base.Theme.AppCompat" 
    android:padding="20sp"> 
 
 
    <TextView 
        android:layout_width="wrap_content" 
        android:layout_height="wrap_content" 
        android:textAppearance="?android:attr/textAppearanceLarge" 
        android:text="Large Text" 
        android:id="@+id/titleHeavyMetal" 
        android:layout_alignParentTop="true" 
        android:layout_centerHorizontal="true" 
        android:textSize="60sp" 
        android:textStyle="bold" 
        android:textColor="#ffffffff" /> 
 
    <TextView 
        android:layout_width="match_parent" 
        android:layout_height="match_parent" 
        android:id="@+id/textHeavyMetal" 
        android:layout_centerHorizontal="true" 
        android:layout_below="@+id/titleHeavyMetal" 
        android:text="Line1: \n-Line2\n-Line3\n-Line4\n-Line5\n-Line6\n-Line7\n-Line8\n-Line9\n-Line10\-Line11\n-Line12\n-Line13\n-Line14 " 
        android:textSize="20sp" 
        android:textColor="#ffffffff" 
        android:layout_marginTop="20dp" 
        /> 
</RelativeLayout>
```
