# Supplementary material for: A paper-based, cell-free biosensor system for the detection of heavy metals and date rape drugs
Source: PLoS One. 2019 Mar 6;14(3):e0210940. doi: 10.1371/journal.pone.0210940 (PMC6402643; doi:10.1371/journal.pone.0210940)
Supplement: S2 File — (ZIP) [file pone.0210940.s016.zip › exportToHTMLres/layout/index.html]

layoutactivity\_main.xml  
activity\_results.xml  
simple\_list\_item.xml  
activity\_analysis.xml  
activity\_take\_photo.xml  
activity\_instructions.xml  
fragment\_heavy\_metals.xml  
activity\_contamination\_list.xml  
activity\_heavy\_metals\_details.xml  
fragment\_heavy\_metals\_details.xml  
