# Supplementary material for: A paper-based, cell-free biosensor system for the detection of heavy metals and date rape drugs
Source: PLoS One. 2019 Mar 6;14(3):e0210940. doi: 10.1371/journal.pone.0210940 (PMC6402643; doi:10.1371/journal.pone.0210940)
Supplement: S2 File — (ZIP) [file pone.0210940.s016.zip › exportToHTMLres/layout/simple_list_item.xml.html]

simple\_list\_item.xml


|  |
| --- |
| simple\_list\_item.xml |

```
<?xml version="1.0" encoding="utf-8"?> 
    <TextView  xmlns:android="http://schemas.android.com/apk/res/android" 
        android:id="@android:id/text1" 
        android:layout_width="match_parent" 
        android:layout_height="wrap_content" 
        android:layout_gravity="center_vertical" 
        android:padding="10dp" 
        android:textSize="30sp" 
        android:textColor="#ffffffff" 
 
        > 
    </TextView>
```
