# Supplementary material for: A paper-based, cell-free biosensor system for the detection of heavy metals and date rape drugs
Source: PLoS One. 2019 Mar 6;14(3):e0210940. doi: 10.1371/journal.pone.0210940 (PMC6402643; doi:10.1371/journal.pone.0210940)
Supplement: S2 File — (ZIP) [file pone.0210940.s016.zip › exportToHTMLres/menu/index.html]

menumenu\_main.xml  
menu\_results.xml  
menu\_analysis.xml  
menu\_take\_photo.xml  
menu\_instructions2.xml  
menu\_contamination\_list.xml  
menu\_heavy\_metals\_details.xml  
