# Supplementary material for: A paper-based, cell-free biosensor system for the detection of heavy metals and date rape drugs
Source: PLoS One. 2019 Mar 6;14(3):e0210940. doi: 10.1371/journal.pone.0210940 (PMC6402643; doi:10.1371/journal.pone.0210940)
Supplement: S2 File — (ZIP) [file pone.0210940.s016.zip › exportToHTMLres/menu/menu_results.xml.html]

menu\_results.xml


|  |
| --- |
| menu\_results.xml |

```
<menu xmlns:android="http://schemas.android.com/apk/res/android" 
    xmlns:app="http://schemas.android.com/apk/res-auto" 
    xmlns:tools="http://schemas.android.com/tools" tools:context="de.anna.cellfreestick.Results"> 
    <item android:id="@+id/action_settings" android:title="@string/action_settings" 
        android:orderInCategory="100" app:showAsAction="never" /> 
</menu>
```
