# Supplementary material for: A paper-based, cell-free biosensor system for the detection of heavy metals and date rape drugs
Source: PLoS One. 2019 Mar 6;14(3):e0210940. doi: 10.1371/journal.pone.0210940 (PMC6402643; doi:10.1371/journal.pone.0210940)
Supplement: S2 File — (ZIP) [file pone.0210940.s016.zip › exportToHTMLres/values/dimens.xml.html]

dimens.xml


|  |
| --- |
| dimens.xml |

```
<resources> 
    <!-- Default screen margins, per the Android Design guidelines. --> 
    <dimen name="activity_horizontal_margin">16dp</dimen> 
    <dimen name="activity_vertical_margin">16dp</dimen> 
</resources>
```
