# Supplementary material for: A paper-based, cell-free biosensor system for the detection of heavy metals and date rape drugs
Source: PLoS One. 2019 Mar 6;14(3):e0210940. doi: 10.1371/journal.pone.0210940 (PMC6402643; doi:10.1371/journal.pone.0210940)
Supplement: S2 File — (ZIP) [file pone.0210940.s016.zip › exportToHTMLres/values/strings.xml.html]

strings.xml


|  |
| --- |
| strings.xml |

```
<resources> 
    <string name="app_name">CellfreeStick</string> 
 
    <string name="hello_world">Hello world!</string> 
    <string name="buttonInstruction">Instructions</string> 
    <string name="buttonTakePhoto">Take Photo</string> 
    <string name="buttonResults">Results</string> 
    <string name="buttonTakePhotoInstruction"> 5.Take Photo</string> 
    <string name="buttonAnalysis">Show Analysis</string> 
    <string name="action_settings">Settings</string> 
    <string name="imageLogo">Bild</string> 
    <string name="textInstruction1">1. Add clean water to test strip 1</string> 
    <string name="textInstruction2">2. Add your sample to test strip 2</string> 
    <string name="textInstruction3">3. Wait 30 min</string> 
    <string name="textInstruction4">4. Put both test strips in the box, test strip 1 on top and test strip 2 on bottom</string> 
    <string name="textInstruction5">5. take photo</string> 
    <string name="textViewResults">Details</string> 
    <string name="analysis">After the user laid its smartphone onto the box and photographed it, the app finds some pixels in the center of each spot 
       and calculates the median of the green values of these pixels. It determines the presence of a contamination in the water by comparing the obtained values for the actual sample and the negative control. 
    Furthermore it also checks the intensity of the positive control to allow an assessment of the functionality of the test. Comparison of the two positive controls allows the calculation of correction factor used to 
    eliminate negative influences on the cell extract by toxic metals or other contaminants.</string> 
 
<!-- TODO: Remove or change this placeholder text --> 
    <string name="hello_blank_fragment">Hello blank fragment</string> 
    <string name="title_activity_instructions2">Instructions</string> 
    <string name="title_activity_take_photo">Photo</string> 
    <string name="title_activity_results">Details</string> 
    <string name="title_activity_analysis">Analysis</string> 
    <string name="title_activity_heavy_metals_details">More Details</string> 
    <string name="title_activity_contamination_list">Results</string> 
 
    <string name="date_rape_drugs">You might want to buy a new drink. Although GHB is applied as anesthetic in medical settings, GHB is frequently misused as date rape drug. It can cross the blood brain barrier, and subsequently can result in various mental and physical obstructions.  Due to its numbing effects, criminals use GHB to rob or abuse. A main problem is the lack of technical equipment in medical centers, preventing to quickly analyse if a patient was intoxicated. Analysis has to take place quickly because it takes less than 12 h for GHB to degrade into non-detectable substances within the body.</string> 
    <string name="Arsenic">Arsenic is present in pesticides, fungicides and metal smelters. The permissible level is 0.02 mg/l. Arsenic can cause bronchitis, dermatitis and poisoning. \n \n Reference: Singh et al., Heavy Metals and living systems: An overview, doi:10.4103/0253-7613.81505</string> 
    <string name="Chromium">Major sources for contaminations with chromium are mines and mineral sources. The permissible level 0.05 mg/l. Chromium can cause damage to the nervous system, fatigue and irritability. \n \n 
        Reference: Singh et al., Heavy Metals and living systems: An overview, doi:10.4103/0253-7613.81505</string> 
    <string name="Nickel">Wrong tapware is the main reason for nickel pollution in tap water. The permissible level is 1 mg per cubic metre. Prolonged exposure to nickel can cause dermatitis and ichting of the skin. \n \n 
     References:  \n http://www.cdc.gov/niosh/docs/81-123/pdfs/0445.pdf, checked on 13th september 2015 
        \n http://2015.igem.org/Team:Bielefeld-CeBiTec/Project/HeavyMetals, checked on 13th september 2015.</string> 
    <string name="Lead">Possible origins of contaminations with lead are paints, pesticides, smoking, automobile emission, mining and burning of coal. The permissible level is 0.1 mg/l. Physical effects can be: mental retardation in children, developmental delay, fatal infant encephalopathy, congenital paralysis, sensor neural deafness and acute or chronic damage to the nervous system, epilepticus, liver, kidney, gastrointestinal damage. 
        \n \n Reference: Singh et al., Heavy Metals and living systems: An overview, doi:10.4103/0253-7613.81505</string> 
    <string name="Copper">Sources of copper contamination are mining, pesticide production, chemical industry and metal piping. The permissible level is 0.1 mg/l. Copper can cause anemia, liver and kidney damage and stomch and intestinal irritation. \n \n 
        Reference: Singh et al., Heavy Metals and living systems: An overview, doi:10.4103/0253-7613.81505</string> 
    <string name="Mercury">Mercury is used in the fabrication process of paper, batteries and pesticides. Its permissible level is 0.01 mg/l. Intoxication results in tremors, gingivitis, minor psychological changes, acrodynia characterized by pink hands and feet, spontaneous abortion, damage to nervous system, protoplasm poisoning. \n \n 
        Reference: Singh et al., Heavy Metals and living systems: An overview, doi:10.4103/0253-7613.81505</string> 
 
 
 
 
</resources>
```
