# Supplementary material for: A paper-based, cell-free biosensor system for the detection of heavy metals and date rape drugs
Source: PLoS One. 2019 Mar 6;14(3):e0210940. doi: 10.1371/journal.pone.0210940 (PMC6402643; doi:10.1371/journal.pone.0210940)
Supplement: S2 File — (ZIP) [file pone.0210940.s016.zip › exportToHTMLres/values/styles.xml.html]

styles.xml


|  |
| --- |
| styles.xml |

```
<resources> 
 
    <!-- Base application theme. --> 
    <style name="AppTheme" parent="Base.Theme.AppCompat.Light.DarkActionBar"> 
        <item name="android:actionBarStyle">@style/ActionBar</item> 
    </style> 
    <style name="ActionBar" parent="Base.Theme.AppCompat.Light.DarkActionBar"> 
        <item name="android:icon">@drawable/icon_igem</item> 
    </style> 
</resources>
```
