# Supplementary material for: A paper-based, cell-free biosensor system for the detection of heavy metals and date rape drugs
Source: PLoS One. 2019 Mar 6;14(3):e0210940. doi: 10.1371/journal.pone.0210940 (PMC6402643; doi:10.1371/journal.pone.0210940)
Supplement: S2 File — (ZIP) [file pone.0210940.s016.zip › exportToHTMLres/values-w820dp/dimens.xml.html]

dimens.xml


|  |
| --- |
| dimens.xml |

```
<resources> 
    <!-- Example customization of dimensions originally defined in res/values/dimens.xml 
         (such as screen margins) for screens with more than 820dp of available width. This 
         would include 7" and 10" devices in landscape (~960dp and ~1280dp respectively). --> 
    <dimen name="activity_horizontal_margin">64dp</dimen> 
</resources>
```
